# Core outcomes for assessing surgical learning curves in high-grade glioma surgery: a European Delphi study

**DOI:** 10.1016/j.bas.2026.106097

**Published:** 2026-05-16

**Authors:** Céline L.G. Neutel, Valerie Diederen, Jiri Bartek, Gerjon Hannink, Maroeska M. Rovers, Mark ter Laan, the Expert Meeting Group, Johnny Duerinck, Steven De Vleeschouwer, Tomas Kazda, Alessia Pallerino, Michael Veldeman, Asgeir S. Jakola, Kostas N. Fountas, Sebastian Pavel, Dan-Andrei Mitrea

**Affiliations:** a Department of Neurosurgery, Universitair Ziekenhuis Brussel (UZ Brussel), Vrije Universiteit Brussel, Brussels, Belgium; b Department of Neurosurgery, University Hospitals Leuven, Belgium; c Department of Neurosciences, Leuven Brain Institute, KU Leuven, Belgium; d Department of Radiation oncology, Masaryk Memorial, Cancer Institute, Brno, Czech Republic; e Department of Neuroscience "Rita Levi Montalcini", University and City of Health and Science Hospital, Turin, Italy; f Department of Neurosurgery, RWTH Aachen University Hospital, Aachen, Germany; g Institute of Neuroscience and Physiology, Department of Clinical Neuroscience, University of Gothenburg, Gothenburg, Sweden; h Region Västra Götaland, Sahlgrenska University Hospital, Department of Neurosurgery, Gothenburg, Sweden; i Department of Neurosurgery, Faculty of Medicine, School of Health Sciences, University of Thessaly, Larisa, Greece; j Brain Institute, Monza Hospital, Bucharest, Romania; k Neuroaxis - Neurology Clinic, Bucharest, Romania; l Department of Neurosurgery, Radboud university medical center, Nijmegen, the Netherlands; m Department of Neurosurgery and Clinical Neuroscience, Karolinska University Hospital and Karolinska Institutet, Stockholm, Sweden; n Department of Medical Imaging, Radboud university medical center, Nijmegen, the Netherlands

**Keywords:** Delphi study, Expert-consensus, Core outcomes, Learning curve, High-grade glioma (HGG)

## Abstract

**Introduction:**

Surgical resection plays an important role in the current treatment of high-grade glioma (HGG). As neurosurgical techniques advance, evaluating the surgical learning curve is important to optimize patient outcomes, ensure safety, and support robust clinical research. However, there is currently no consensus on which outcome measures should be used to evaluate the surgical learning curve.

**Research question:**

What are the most appropriate outcome measures to assess the surgical learning curve in HGG surgery?

**Material and methods:**

A three-round Delphi study comprising two online questionnaires and an expert consensus meeting was conducted. Outcomes were scored on relevance and feasibility on a 9-point Likert scale. Respondents included European neurosurgeons, neuro-oncologists, radiation oncologists, medical oncologists, and specialized nurses. The consensus meeting involved 13 experts, including two high-grade glioma patients.

**Results:**

The first and second questionnaires consisted of 24 and 27 (of which 22 were reassessed) outcomes and received 50 and 64 responses, respectively. Following the two questionnaire rounds, eight outcomes were initially accepted; these were subsequently reviewed during the expert consensus meeting, which led to the establishment of a final set of five outcomes, namely: percentage tumor resected, residual tumor remnant, permanent post-operative new neurological symptoms/deterioration, adverse events Clavien-Dindo ≥2 < 72 h after surgery and onco-functional outcome scale.

**Discussion and conclusion:**

This study provides the first consensus-based set of outcome measures for assessing the learning curve in HGG surgery, providing a foundation for standardized assessment of learning curves in HGG surgery. It is now necessary to validate this set in research practice.

## Highlights

• Three-round Delphi process including questionnaires and expert consensus meeting.
• European expert consensus on core outcomes for measuring HGG surgery learning curve.
• Final set of five core outcomes to assess learning curve in HGG surgery.

## 1 Introduction

Research into learning curves has gained increasing recognition and interest in surgical practice (Hopper et al., 2007; McCulloch et al., 2009; Wijburg et al., 2022; Brenkman et al., 2023). The learning curve signifies the process of mastering a surgical procedure over time, with the implicit understanding that proficiency improves with experience (Subramonian and Muir, 2004). Particularly in light of the rapid emergence of new technologies, monitoring the surgical learning curve is important for ensuring patient safety and quality control (McCulloch et al., 2009; Taylor et al., 2025). Currently, the most optimal treatment of high-grade glioma (HGG) consists of safe and maximal surgical resection, when feasible, followed by chemoradiation. Since the extent of tumor resection is correlated with the survival of patients (Stummer et al., 2008; Goldbrunner et al., 2025), advances in glioblastoma surgery has mainly been driven by innovations in surgical techniques and technology but also by our evolving understanding of HGG pathobiology (Schupper et al., 2021). As neurosurgeons endeavor to enhance patient outcomes, optimize surgical procedures, and minimize complications, the "learning curve" and outcome standardization concept deserves increased attention within this field.

Assessing the learning curve in HGG surgery not only aids in benchmarking surgeons progress, enhancing patient safety and improvement of patient outcomes, but also in guiding surgical training, optimizing resource allocation, driving quality improvement, and ultimately providing the best possible care for patients. Assessing the surgical learning curve also plays an important role in scientific research and the evaluation of (new) surgical techniques within clinical trials (McCulloch et al., 2009). If the effects of a (new) surgical procedure are evaluated before the surgeon has reached the desired level of stable performance, the true impact of the intervention may be misrepresented (Ergina et al., 2013).

However, quantifying and evaluating this learning curve needs the identification of a “core” set of outcomes that can objectively assess a surgeon's progression. Broadly speaking, there are two groups of measurable variables in this context, namely patient outcome measures and surgery related factors (Tekkis et al., 2005). It is important to recognize that the relevant outcome for assessing the surgical learning curve may vary for each type of surgery (Hopper et al., 2007). For example, in the context of HGG care, while the extent of resection significantly influences patient prognosis, the nature of the disease itself and the impact of adjuvant treatment also play pivotal roles in this regard. Questions may thus arise about whether survival is the optimal variable to consider when examining the learning curve and performance of a neurosurgeon. It is therefore important to critically define and clearly delineate these crucial outcome measures in advance.

In 2023, Retzer et al. (2023) published an extensive mixed-method study determining a Core Outcome Set (COS) for glioma treatment. Although this study covers the vast majority of key outcomes for glioma care in general, there is no specification regarding important outcomes related to the surgical learning curve. The primary aim of this study therefore is to achieve (European) consensus on the most important outcome measures for assessing the learning curve in HGG surgery. By leveraging the experiences of experts in the field, we intend to arrive at a comprehensive set of outcome measures that can serve as the basis for evaluating the learning curve and guiding further research and practice in HGG surgery.

## 2 Methods

To develop the outcome set, the RAND/UCLA Appropriateness Method, a modified Delphi process was used (Kathryn et al., 2001). Reporting of this Delphi process was conducted in accordance with the DELPHISTAR guidelines for standardized reporting of Delphi studies (Niederberger et al., 2024). The number of rounds was defined in advance. The study was conducted in three phases: two online questionnaire rounds in which stakeholders across Europe rated the outcomes and an expert consensus meeting to come to a final short list of outcomes.

### 2.1 Review of literature and drafting first questionnaire

The initial questionnaire, containing a broad list of potentially relevant outcomes for assessing the surgical learning curve in HGG surgery, was developed based on a comprehensive review of the literature and expert opinion. This review aimed to synthesize current knowledge on outcome measures commonly used to evaluate surgical learning curves, with a particular focus on neurosurgery. The literature search was kept broad and was conducted in the PubMed and Embase databases using the terms “glioblastoma,” “high-grade glioma,” “surgery,” and “learning curve.”

The primary objective of this process was to inform respondents of the current state of evidence and expert opinion on outcome measures for surgical learning curves, thereby establishing a foundation for the first online questionnaire.

### 2.2 First delphi questionnaire

The questionnaire (supplementary item 1) was developed and sent out using the web-based application LimeSurvey (LimeSurvey GmbH, n.d.). Participants were instructed to complete the questionnaire independently, without consulting other respondents. All questionnaires were completed anonymously, unless the respondent voluntarily provided an email address. All questionnaires from this Delphi study started with demographic questions to obtain insight into the characteristics of the respondents. Participants were asked about their current profession and country of employment, their experience in neuro-oncology, and whether they are affiliated with a policy-making body. Following these initial questions, the main section of the questionnaire started, in which the outcomes were assessed. The outcome list for the first questionnaire was designed to be as comprehensive as possible, capturing a broad range of perspectives. Outcomes were grouped as stated below:

1. Operative outcomes
  • General operative outcomes
  • Surgeon-level outcomes
  • Organization-level outcomes
2. Patient outcomes
  • General patient outcomes
  • Adverse events
  • Mortality
  • Functioning
3. Patient reported outcomes

Each outcome was evaluated based on two criteria: relevance, defined as the extent to which the outcome reflects the neurosurgical learning curve, and feasibility, referring to the practicality of measuring the outcome in routine clinical practice. Ratings were assigned using a 9-point Likert scale, where 1 indicated “definitely not relevant” or “definitely not feasible” and 9 indicated “highly relevant” or “highly feasible”. Additionally, respondents were able to leave comments regarding the outcomes and were invited to propose any outcomes they believed had been missed by the study team.

### 2.3 Dissemination of the delphi questionnaires

The first Delphi questionnaire was distributed to stakeholders across Europe. Targeted participants included neurosurgeons, neuro-oncologists, radiation oncologists, medical oncologists, specialized neuro-oncology nurses, and physician assistants. We aimed to include specialists from all neuro-oncological disciplines to obtain broad input. Respondents were asked to indicate whether they were explicitly specialized in neuro-oncology (excluding general residency but including fellowships) to assess their suitability. The questionnaires were disseminated via email through the European Association of Neurosurgical Societies (EANS), the Dutch Neuro-Oncological Society (LWNO), and the research team's professional networks. Recipients were encouraged to forward the questionnaire link to relevant colleagues within their institutions or networks.

### 2.4 Analysis of the first delphi questionnaire and development of the second delphi questionnaire

After the first questionnaire round, the answers of the respondents were processed and analyzed. Responses were classified into three categories: scores of 1–3 were considered “irrelevant” or “unfeasible”, scores of 4–6 were considered “potentially relevant” or “potentially feasible”, and scores of 7–9 were considered “relevant” or “feasible”. Outcomes were classified as accepted if ≥ 75% of respondents rated them as both relevant and feasible. Conversely, outcomes were discarded if ≥ 75% rated them as both irrelevant and unfeasible. Outcomes that were neither accepted nor discarded were carried forward to the subsequent questionnaire. If ≥ 75% of respondents rated an outcome as relevant but there was no consensus on feasibility, or vice versa, the outcome was also carried forward. In such cases, the outcome was reassessed in the following round solely on the aspect (i.e., relevance or feasibility) for which consensus had not been achieved. For each outcome carried forward, the median score and interquartile range (IQR) from the first round were presented to participants in the second questionnaire round, providing insight into how other respondents had evaluated these outcomes. Additional outcomes identified by respondents in the first questionnaire were incorporated into the second questionnaire. Comments from the first questionnaire were condensed and incorporated into the corresponding outcome descriptions in the second questionnaire. This was done to inform participants of the second round about the perspectives and considerations expressed by others.

For the second questionnaire (supplementary item 2), outcomes were grouped as follows to maintain clarity and facilitate respondent engagement:

1. Outcomes tested for relevance and feasibility
2. Outcomes tested for relevance
3. Outcomes tested for feasibility

Again, respondents were encouraged to propose any outcomes they believed had been missed by the study team.

### 2.5 Analysis of the second delphi questionnaire

The answers of the second questionnaire were processed and analyzed in the same manner as the answers of the first questionnaire round. All outcomes were carried forward to the Delphi Expert Consensus meeting.

### 2.6 Identifying relevant experts for the Delphi Expert Consensus meeting

In selecting participants, attention was given to achieving a balanced representation in terms of professional background and country of practice. Experts invited to the consensus meeting were selected from respondents of the first questionnaire round, who were explicitly specialized in neuro-oncology, had provided their email address, and expressed interest in participating in the meeting. A meeting date was scheduled in coordination with these experts. In addition, several experts from the second questionnaire round who had provided their contact details were also invited to participate. Two HGG patients from two different countries were invited to participate in the meeting. Selection criteria for patients included a confirmed diagnosis of glioma, sufficient proficiency in English, and the cognitive ability to understand the material and actively participate in the discussion as assessed by their treating neurosurgeon and the coordinating researcher. In the following, the term “experts” refers to both the participating medical specialists and the two participating patients.

### 2.7 Delphi Expert Consensus meeting

The digital expert consensus meeting was held using Zoom (Zoom Video Communications, Inc., San Jose, California, U.S.). The aim of the meeting was to review, discuss and validate the results of the questionnaires and to come to a consensus-based list of outcomes for measuring the neurosurgical learning curve in HGG surgery. An independent senior researcher, experienced in chairing consensus meetings, moderated the session. The meeting was recorded to ensure accurate documentation and allow verification of the consensus process.

One week prior to the final online expert consensus meeting, the results of the two questionnaire rounds were distributed to all participating experts to facilitate their preparation. At the start of the meeting, all results from both rounds were presented again to ensure a shared understanding. Subsequently, the outcomes that had been accepted based on both relevance and feasibility were discussed in detail. Each outcome was addressed individually, followed by a formal vote. Outcomes were accepted if more than 50% of the participants voted in favor.

After the discussion of all outcomes previously accepted in the questionnaires, experts were given the opportunity to propose additional outcomes for discussion, such as those that had been accepted for relevance but not for feasibility or vice versa.

A second expert meeting was convened five weeks after the first to discuss an outcome not previously addressed, and it was conducted in the same manner as described for the first meeting.

## 3 Results

**Fig. 1:**
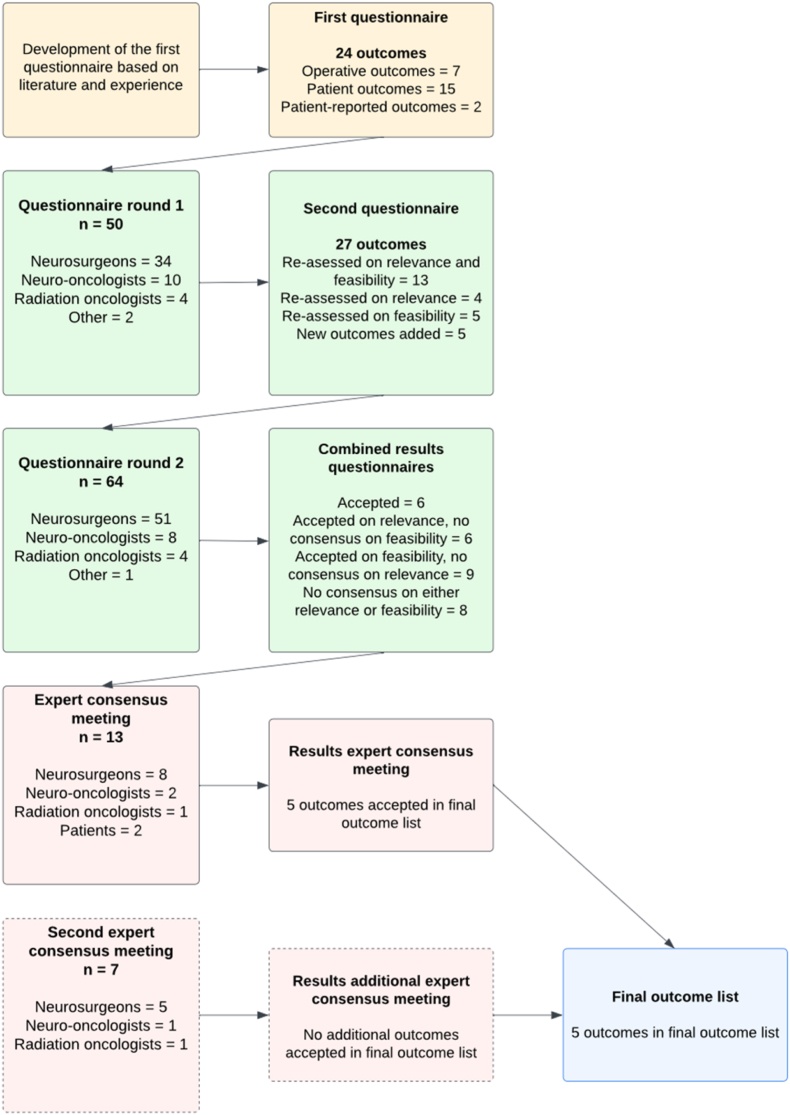
overview development of outcome set.

An overview of the study outline and study results is depicted in Fig. 1.

### 3.1 First delphi questionnaire

A total of 24 outcomes were taken up into the first questionnaire. Seven outcomes were grouped as surgery related outcomes, 15 as patient outcomes and two as patient-reported outcomes. The first questionnaire is available as supplementary item 1.

**Fig. 2:**
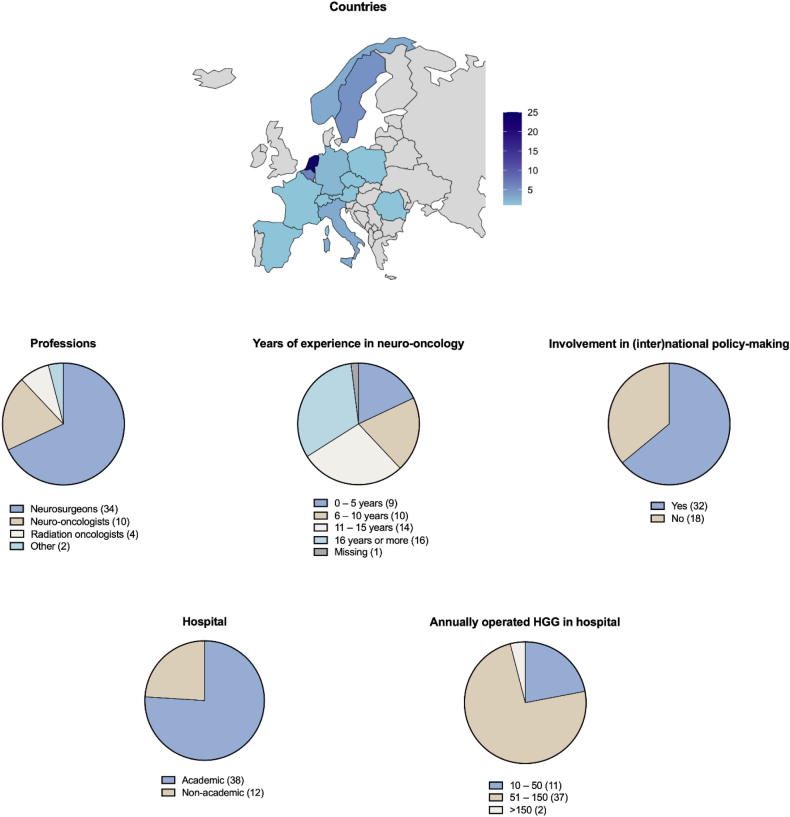
Respondent demographics first questionnaire round.

The questionnaire was sent on the 27th of November 2024 and by the 20th of January 2025 a total of 50 responses were collected. The demographic characteristics of the respondents are shown in Fig. 2.

Following analysis, two outcomes were classified as both relevant and feasible and were therefore directly carried forward to the expert consensus meeting. No outcomes were classified as irrelevant and unfeasible; consequently, none were discarded based on the first questionnaire round. Five outcomes reached consensus on relevance but not on feasibility, while four outcomes reached consensus on feasibility but not on relevance. An additional 13 outcomes did not reach consensus on either relevance or feasibility. The results of the first questionnaire is available in supplementary item 3. A total of five new outcomes were proposed by the respondents of the first questionnaire and were thus added to the second questionnaire.

### 3.2 Second delphi questionnaire

The second questionnaire consisted of 27 outcomes. Thirteen outcomes were tested again on both relevance and feasibility, four were tested again only on relevance and five outcomes were only tested again on feasibility. Five outcomes, proposed by respondents from the first round, were added to the second questionnaire and thus tested on both relevance and feasibility. The second questionnaire is available as supplementary item 2.

**Fig. 3:**
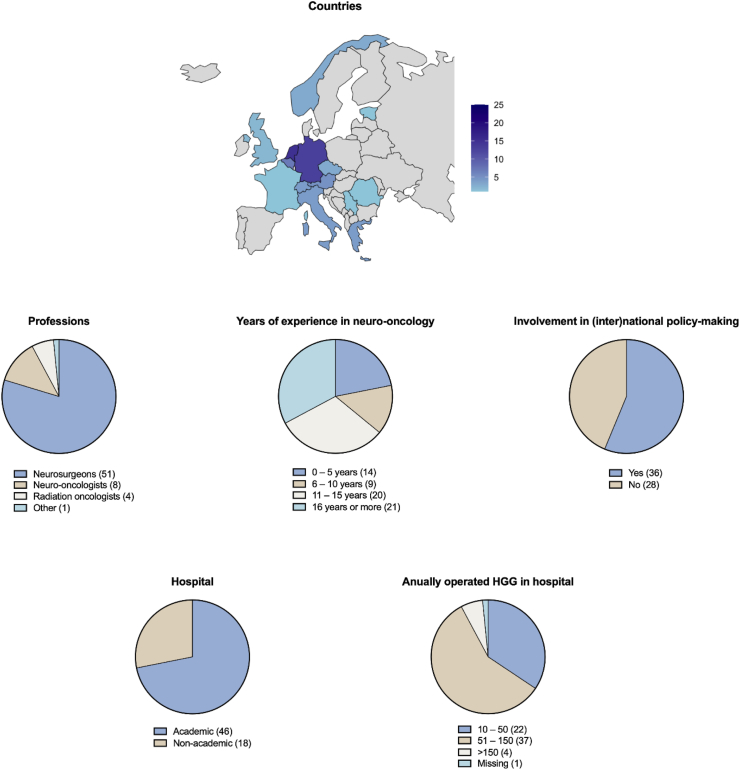
Respondent demographics second questionnaire round.

The questionnaire was sent on the 7th of March 2025 and was closed on the 21st of May 2025. A total of 70 responses were collected. Six responses were excluded from the analysis. The reasons for exclusion were as follows: two participants did not specialize in neuro-oncology; one participant assigned a score of nine to every outcome for both relevance and feasibility, raising concerns about the validity of the response; one participant was from a non-European country; and two questionnaires were submitted by the same individual but differed from each other, making it impossible to determine which response should be considered. Thus 64 responses remained for analyses. The demographic characteristics of the respondents are shown in Fig. 3.

After the two questionnaire rounds, a total of eight outcomes were classified as both relevant and feasible. Again, no outcomes were classified as irrelevant and unfeasible. Six outcomes reached consensus on relevance but not on feasibility, and nine outcomes reached consensus on feasibility but not on relevance. Eight outcomes did not reach consensus on either relevance or feasibility. No new outcomes were proposed by respondents of the second questionnaire.

**Fig. 4:**
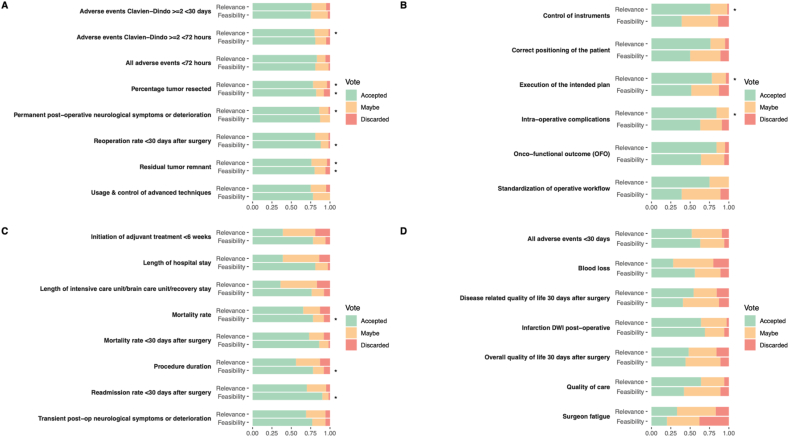
Combined results of the first and second questionnaires. A: outcomes accepted for both relevance and feasibility; B: outcomes accepted for relevance but lacking consensus on feasibility; C: outcomes accepted for feasibility but lacking consensus on relevance; D: outcomes without consensus on either relevance or feasibility. Scores marked with an asterisk indicate outcomes that reached consensus in the first questionnaire round and were directly carried over to the combined results without re-evaluation in the second round.

The final results of the questionnaire rounds are shown in Fig. 4. The full table is available as supplementary item 4.

### 3.3 Expert Consensus Meeting

For the third and final round of this Delphi study, 13 experts participated in the online expert consensus meeting. The panel comprised eight neurosurgeons, two neuro-oncologists, one radiation oncologist, and two patients, with participants representing eight different European countries. The demographic characteristics of the expert group are available as supplementary item 5.

Of the eight outcomes previously accepted through the Delphi questionnaires, four were reaffirmed and retained during the expert consensus meeting. However, three outcomes that had initially met the criteria for inclusion were ultimately excluded during the meeting: *“Re-operation rate within 30 days after surgery”*, *“All adverse events <72 h after surgery”* and *“Adverse events classified as Clavien-Dindo grade ≥2 < 30 days after surgery”*. The first was excluded due to its low incidence, which was therefore considered insufficiently sensitive for capturing variations in the learning curve. Additionally, it was regarded as overlapping with the already accepted outcome “*Adverse events classified as Clavien-Dindo grade ≥2 < 72 h after surgery”*, given that re-operations are classified as adverse events. The outcome *“All adverse events <72 h after surgery”* was considered too broad, particularly in light of the inclusion of *“Adverse events classified as Clavien-Dindo grade ≥2 < 72 h after surgery”* in the outcome set. Lastly, the outcome *“Adverse events classified as Clavien-Dindo grade ≥2 < 30 days after surgery”* was excluded due to the overlap with the outcome *“Adverse events classified as Clavien-Dindo grade ≥2 < 72 h after surgery”*. Extending the time frame from 72 h to 30 days was considered less appropriate, as it could include adverse events potentially attributable to factors unrelated to the surgical procedure itself, such as early disease progression.

In addition to outcomes accepted through the questionnaire rounds, one further outcome, namely *“Onco-functional Outcome Class”* (OFO) (Gerritsen et al., 2024), was reintroduced for discussion at the expert meeting. The OFO is derived by combining extent of resection with the functional outcome assessed approximately 6 weeks postoperatively. Gross total resection (GTR) was defined as the absence of residual contrast-enhancing tumour on early postoperative MRI, whereas any visible residual enhancing disease was classified as subtotal resection (STR). Functional deterioration is defined as a decrease in KPS (≥10 points) and/or an increase in NIHSS (≥1 point). Based on these two dimensions, patients can be categorised into four groups: OFO 1 (GTR without deterioration), OFO 2 (STR without deterioration), OFO 3 (GTR with deterioration), and OFO 4 (STR with deterioration). Although accepted based on relevance but discarded based on feasibility in the second questionnaire round, the experts present considered it highly relevant due to its straightforward implementation and its capacity to capture both clinical and technical aspects of surgical outcomes both at each individual patient's level and at a group level. The two patient representatives also expressed strong support for this outcome, emphasizing its importance from their perspective since it aligns simultaneously with the two concerns patients value most: *“how long will I live?”* and *“in which (neurological) condition will this be?”*. On the premise that the previously low feasibility rating was driven by the novelty of the outcome rather than true concerns about its practical applicability, the expert panel decided to include it in the final list of outcomes. The results of the expert consensus meeting are shown in Table 1.

**Table 1:** Results of expert consensus meeting.

Outcomes accepted in final list	Vote in^b^	Domain
Percentage of tumor resected^c^	64%	Surgical performance
Residual tumor remnant^c^	64%	Surgical performance
Permanent^a^ post-operative new neurological symptoms or deterioration	83%	Neurological function
Adverse events Clavien-Dindo ≥2 < 72 h after surgery	83%	Adverse events
Onco-functional outcome scale (OFO)	82%	Surigal performance and neurological function
**Outcomes discarded during the meeting**	**Vote in**^b^	
Re-operation rate <30 days after surgery	33%	
All adverse events <72 h after surgery	8%	
Adverse events Clavien-Dindo ≥2 < 30 days after surgery	0%	

a Permanent is defined as still present at 30 days after the procedure.

b A threshold of 50% was used to define consensus during the expert meeting and thus to accept or reject an outcome taking into consideration both relevance and feasibility.

c Measured using MRI-based volumetric assessment.

### 3.4 Second expert consensus meeting

An additional expert meeting was convened five weeks after the initial session to address the outcome “*Usage and control of advanced techniques*” which had not been discussed during the first Expert Consensus Meeting, despite meeting the predefined criteria for discussion. Despite its acknowledged relevance, the outcome was excluded from the final list due to limited support (only 29% voted to include the outcome) and concerns regarding definition, availability, and measurability (see Supplementary item 6 for details).

## 4 Discussion

This study is the first to define a set of outcome measures specifically for the assessment of the neurosurgical learning curve in HGG surgery. By establishing these outcomes a priori, future analyses of learning curves can be conducted in a standardized manner.

The final outcome set comprises five outcomes, i.e percentage tumor resected, residual tumor remnant, permanent post-operative new neurological symptoms or deterioration, adverse events Clavien-Dindo ≥2 < 72 h after surgery and onco-functional outcome scale. Two outcomes pertain to the domain of surgical performance, one to neurological function, one to adverse events, and one is relevant to both neurological function and surgical performance. We consider this set to provide a comprehensive yet focused representation of key parameters for evaluating the neurosurgical learning curve, each reflecting a slightly distinct aspect of the surgical procedure or its results. The inclusion of both extent of resection and residual tumour remnant reflects the current lack of consensus on which parameter is most prognostically relevant in high-grade glioma surgery. Although closely related, they are not interchangeable and were therefore both retained, with extent of resection also incorporated in the composite OFO score. Similarly, while permanent neurological deficits may be perceived as part of the Clavien–Dindo classification, this system captures a broader range of complications and primarily reflects the type of intervention required to treat a complication, rather than the complication itself or its specific clinical manifestation. Given its clinical relevance, neurological function was therefore considered to warrant separate reporting. Further validation may identify overlap between measures, potentially allowing refinement of the set.

Experts emphasized the importance of maintaining a concise set of outcomes and prioritizing those already integrated into standard care or routine follow-up protocols, in order to maximize feasibility for both research and clinical implementation. This insight was also reflected in both the results and the comments obtained from the two questionnaire rounds. One outcome, being the OFO score, that was ultimately included in the final list by the expert panel received a low feasibility score in the second questionnaire round. Assuming that the previously low feasibility score reflected the outcome's novelty rather than a genuine concern about its feasibility, the expert panel decided to include it in the final list of outcomes. This decision illustrates the distinction between perceived and actual feasibility. Newly introduced outcome measures, such as the OFO score, may initially be rated as less feasible because clinicians are unfamiliar with them or have limited experience in applying them (i.e., perceived feasibility), rather than because of true practical barriers. The expert panel considered it likely that, with increasing familiarity and standardisation, the OFO score would become a feasible outcome in clinical practice. In contrast, outcome measures that have been established for a longer period but were still rated as not feasible were interpreted as reflecting genuine practical limitations (i.e., actual feasibility), a view that was confirmed during the consensus meeting. We acknowledge that feasibility is context-dependent and may evolve over time; therefore, re-evaluating outcome measures in future practice remains important.

The decision to exclude several postoperative morbidity-related outcomes, specifically re-operation rate <30 days after surgery and adverse events Clavien-Dindo ≥2 < 30 days after surgery, reflects broader methodological challenges in defining suitable endpoints for learning-curve assessment in HGG surgery. Although 30-day morbidity and reoperation rates are commonly used endpoints in other surgical fields to evaluate performance and learning curves, the expert panel considered these measures less specific in the context of high-grade glioma surgery. In neuro-oncology, postoperative outcomes beyond the immediate perioperative period are strongly influenced by tumour biology, adjuvant therapies, and disease progression. As a result, they are less capable of isolating the effect of surgical performance. Early postoperative outcomes (within 72 h) were therefore deemed more appropriate, as they more directly capture surgery-related morbidity and are more sensitive to variations in surgical technique and experience.

It is important to acknowledge that learning-curve assessment can be conceptualised at different levels, ranging from early technical skill acquisition to broader evaluations of surgical quality over time. Measures such as operative time and blood loss are often particularly sensitive to early-stage learning effects (Wehrtmann et al., 2020), whereas the present consensus prioritised outcomes reflecting oncological efficacy and neurological safety, which were deemed most relevant for assessing performance in high-grade glioma surgery. Consequently, the selected outcome set reflects a focus on clinically meaningful, patient-centered measures rather than early procedural efficiency alone. A different framing of the study, specifically targeting initial skill acquisition, may have resulted in a different selection of endpoints.

This Delphi study has several strengths. It is the first study to systematically define an outcome set specifically to measure the neurosurgical learning curve in HGG surgery, thereby addressing a critical gap in the current literature. The use of the rigorous, multi-round Delphi methodology ensured a transparent and structured approach to gain consensus. The respondents of the questionnaires as well as the expert panel comprised of participants from multiple European countries, capturing diverse clinical practices and perspectives that enhance the generalizability of the findings. Importantly, a multidisciplinary group of specialists was involved throughout the process, including neurosurgeons, neuro-oncologists, and radiation oncologists, ensuring that the selected outcomes were evaluated from different professional perspectives. In addition, two patients participated in the expert meeting, contributing valuable perspectives on the relevance and impact of the outcomes from a patient-centered viewpoint. The study explicitly considered the feasibility of each outcome, with particular attention to whether outcomes are already integrated into standard care or follow-up protocols. By explicitly considering both relevance and feasibility, the resulting outcome set is both scientifically robust and practically applicable which is of importance for future clinical implementation.

This study also has its limitations. First, although the study achieved good representation from multiple European countries, the majority of responses in the first round originated primarily from the Netherlands, and in the second round from the Netherlands and Germany. Despite various initiatives, recruiting a larger number of respondents from other countries remained challenging. Sensitivity analysis of the first questionnaire round showed no notable differences between Dutch and non-Dutch respondents. In the second round, a sensitivity analysis comparing responses from the Dutch and German subgroup (44% of all responses) with those from the remaining countries showed that fewer outcomes reached consensus for inclusion within the Dutch/German subgroup. Several outcomes that did reach consensus for inclusion in the other countries were not included by the Dutch/German subgroup, namely: procedure duration (feasibility), length of ICU/BCU/recovery stay (feasibility), length of hospital stay (feasibility), initiation of adjuvant treatment <6 weeks (feasibility), transient post-operative neurological symptoms or deterioration (feasibility), all adverse events <30 days (feasibility), adverse events Clavien-Dindo≥2 < 30 days (feasibility), infarction on DWI (relevance and feasibility), usage and control of advanced techniques (relevance and feasibility), correct positioning of the patient (relevance), readmission rate <30 days (relevance), mortality rate <30 days (relevance). These results are presented in Supplementary Item 7. Nevertheless, the expert panel in the final consensus meeting was internationally diverse, and all outcomes included in the final set had achieved sufficient consensus in both subgroups of the second questionnaire round, supporting the robustness and international applicability of the final results.

Second, due to the method of questionnaire distribution, through societies and by encouragement to forward the questionnaire within personal networks, it was not possible to calculate an accurate response rate for the questionnaires.

Third, during post-hoc data verification, one respondent from the first questionnaire round was excluded, as he did not meet the eligibility criteria, being specialized in neuro-oncology. We performed a sensitivity analysis and although excluding this respondent led to two minor variations in the construction of the second questionnaire, these had no impact on the final combined results, consensus outcomes, or overall conclusions.

The development of this outcome set lays a foundation for future efforts toward international standardization and benchmarking of neurosurgical training and performance, and may facilitate future research through improved comparability across studies. To further promote the use of this outcome set and facilitate its routine measurement, it could be integrated into the existing Core Outcome Set (COS) for gliomas (Retzer et al., 2023). Routine measurement of these outcomes in both clinical practice and research settings would support and streamline future investigations into neurosurgical learning curves.

Learning curve analysis can be conducted in various ways, with one of the most commonly used approaches being retrospective plotting of data and visually identifying the point at which a plateau in performance is reached. However, other methods for measuring the learning curve might require the definition of threshold values that distinguish between successful and unsuccessful procedures. Facilitating international adoption and potential integration into broader glioma core outcome frameworks will first require further validation of the proposed outcome set. Future work should therefore focus on prospective validation in international multicentre cohorts to assess reliability, reproducibility, and generalizability across different institutional settings and surgical technologies. In addition, studies are needed to establish clinically meaningful threshold values and to evaluate interobserver agreement, particularly for imaging-based and composite outcomes. After validation and potential further refinement, the outcomes may be incorporated into core outcome sets to facilitate broad international implementation.

## 5 Conclusion

Through a structured three-round Delphi process, consisting of two online questionnaires and an expert consensus meeting, this study established European expert consensus and identified a final set of five outcome measures to assess the learning curve in HGG surgery. These accepted measures were percentage tumor resected, residual tumor remnant, permanent post-operative new neurological symptoms or deterioration, adverse events Clavien-Dindo ≥2 < 72 h after surgery and onco-functional outcome scale.

## Authorship contribution

CN: project management, conceptualization, methodology, data collection, data analysis, expert meetings, writing manuscript (original draft).

VD: data collection, data analysis, expert meetings, writing manuscript (review and editing).

JB: conceptualization, methodology, expert meetings, writing manuscript (review and editing).

GH: conceptualization, methodology, data analysis, writing manuscript (review and editing).

MR: conceptualization, methodology, expert meetings, writing manuscript (review and editing), supervision.

MtL: conceptualization, methodology, expert meetings, writing manuscript (review and editing).

JD: expert meetings, feedback, writing manuscript (review and editing).

SDV: expert meetings, feedback, writing manuscript (review and editing).

TK: expert meetings, feedback, writing manuscript (review and editing).

AP: expert meetings, feedback, writing manuscript (review and editing).

MV: expert meetings, feedback, writing manuscript (review and editing).

AJ: expert meetings, feedback, writing manuscript (review and editing).

KF: expert meetings, feedback, writing manuscript (review and editing).

SP: expert meetings, feedback, writing manuscript (review and editing).

DM: expert meetings, feedback, writing manuscript (review and editing).

All authors read and approved the final manuscript.

## Ethical approval

Not applicable.

## Funding sources

Not applicable.

## Declaration of competing interest

The authors declare that they have no known competing financial interests or personal relationships that could have appeared to influence the work reported in this paper.
