# Supplementary material for: Core outcomes for assessing surgical learning curves in high-grade glioma surgery: a European Delphi study
Source: Brain Spine. 2026 May 16;6:106097. doi: 10.1016/j.bas.2026.106097 (PMC13397579; doi:10.1016/j.bas.2026.106097)
Supplement: Multimedia component 1 [file mmc1.pdf]

Supplementary Item 1

Core outcomes for assessing surgical learning curves in high-grade glioma surgery: a European Delphi study

Céline L.G. Neutel, MD[1], Valerie Diederen[1], Jiri Bartek, MD, PhD[2], Gerjon Hannink, PhD[3], Maroeska M. Rovers, PhD[3], Mark ter Laan, MD, PhD[1], the Expert Meeting Group[#]

[1] Department of Neurosurgery, Radboud university medical center, Nijmegen, The Netherlands.

[2] Department of Neurosurgery and Clinical Neuroscience, Karolinska University Hospital and Karolinska Institutet, Stockholm, Sweden

[3] Department of Medical Imaging, Radboud university medical center, Nijmegen, The Netherlands.

#

- Johnny Duerinck, MD, PhD, Department of Neurosurgery, Universitair Ziekenhuis Brussel, Vrije Universiteit Brussel, Brussels, Belgium
- Steven De Vleeschouwer, MD, PhD, Department of Neurosurgery, University Hospitals Leuven, Belgium and Department of Neurosciences, Leuven Brain Institute, KU Leuven, Belgium
- Tomas Kazda, MD, PhD, Department of Radiation oncology, Masaryk Memorial Cancer Institute, Brno, Czech Republic
- Alessia Pellerino, MD, PhD, Department of Neuroscience "Rita Levi Montalcini", University and City of Health and Science Hospital, Turin, Italy
- Michael Veldeman MD PhD, Department of Neurosurgery, RWTH Aachen University Hospital, Aachen, Germany
- Asgeir S. Jakola, MD, PhD, Institute of Neuroscience and Physiology, Department of Clinical Neuroscience, University of Gothenburg, Gothenburg, Sweden and Region Västra Götaland, Sahlgrenska University Hospital, Department of Neurosurgery, Gothenburg, Sweden
- Kostas N. Fountas, MD, PhD, Department of Neurosurgery, Faculty of Medicine, School of Health Sciences, University of Thessaly, Larisa, Greece
- Sebastian Pavel, MD, Brain Institute, Monza Hospital, Bucharest, Romania
- Dan-Andrei Mitrea, MD, Neuroaxis - Neurology Clinic, Bucharest, Romania

Supplementary Item 1

First digital questionnaire

Dear respondent,

Welcome to the first questionnaire of the Delphi study 'Obtaining consensus for assessing surgical learning curves in high-grade glioma

surgery: a European Delphi study.'

The aim of this Delphi study is to achieve consensus on which outcomes we should consider and include for assessing the surgical learning

curve of a neurosurgeon when operating on high-grade gliomas. This is important because neurosurgery is a rapidly evolving field with

innovations emerging at a fast pace. Measuring the surgical learning curve can be valuable in clinical practice, in the training of

neurosurgeons, and within medical research for evaluating new interventions.

Since it is unclear which outcome measures are important for assessing this learning curve, and they may differ from those relevant for

evaluating the effectiveness of a treatment, we have initiated this study. A Delphi study consists of several steps, and this questionnaire is

the first step. We hope to gather input from as many stakeholders in neuro-oncology as possible regarding which outcomes they believe

are important.

Section A: INTRODUCTION AND DEMOGRAPHICS

This Delphi study aims to identify the most relevant outcomes for assessing the learning curve of neurosurgeons. The goal is to determine which aspects of surgical skills, operative organization, and patient outcomes provide the best insight into a neurosurgeon's professional development over time.

We expect that completing this questionnaire will take between 10 and 15 minutes.

Potential outcomes will be presented one by one. Each outcome will be rated on two dimensions on a scale of 1 to 9:

Relevance: "How relevant is it to measure this outcome when assessing a neurosurgical learning curve?" Feasibility: "How feasible is it to consistently measure this outcome for the purpose of assessing a neurosurgical learning curve?"

It is possible to go back to a previous question. To do so, use the "Previous" button at the bottom left of the page, not the "Back" button on your internet browser.

If you have any questions before, during, or after completing the questionnaire, please feel free to contact the research team at celine.neutel@radboudumc.nl

----

Additional information:

The questionnaire consists of three main groups: operative outcomes (divided into general operative outcomes, surgeon-level outcomes, and organization-level outcomes), patient outcomes (divided into general patient outcomes, adverse events, mortality, and functioning), and patient-reported outcomes.

At the end of the questionnaire, we will ask you to create a top 5 of the outcomes that, in your opinion, are the most important for measuring a neurosurgeon's learning curve. There will also be an opportunity for you to suggest additional outcomes that you believe should be considered as well.

A1. What is your age?

- Younger than 25 years ☐
- 25 - 34 years ☐
- 35 - 44 years ☐
- 45 - 54 years ☐
- 55 - 64 years ☐
- 65 years and older ☐

LimeSurvey

A2. In which country do you currently work?

- Albania
- Andorra
- Austria
- Belgium
- Bosnia and Herzegovina
- Bulgaria
- Croatia
- Cyprus
- Czech Republic
- Denmark
- Estonia
- Finland
- France
- Greece
- Germany
- Hungary
- Ireland
- Iceland
- Italy
- Kosovo
- Latvia
- Liechtenstein
- Lithuania
- Luxembourg
- Malta
- Moldova
- Monaco
- Montenegro
- North Macedonia
- Norway
- Poland

LimeSurvey

A3. **What is your current profession?**

- Neurosurgeon ☐
- Neurologist / Neuro-oncologist ☐
- Radiologist ☐
- Radiotherapist ☐
- Specialized nurse ☐
- Other ☐

Other

A4. **Are you working in an academic or non-academic hospital?**

- Academic hospital ☐
- Non-academic hospital ☐

A5. **Approximately how many high-grade gliomas are operated on annually at the center where you work?**

- < 10 ☐
- 10 - 50 ☐
- 51 - 150 ☐
- > 150 ☐
- I don't know ☐

A6. **Do you hold a position in a national working group involved in developing guidelines and/or creating policies related to neuro-oncological care?**

- Yes ☐
- No ☐

A7. How many years of experience do you have in neuro-oncological care?

Please <u>exclude</u> your <u>medical specialist training</u>, and <u>include</u> any <u>fellowships</u>.

- 0 - 5 years ☐
- 6 - 10 years ☐
- 11 - 15 years ☐
- 16 years or more ☐
- Not specialized in neuro-oncology ☐

Section B: 1. OPERATIVE OUTCOMES: General operative outcomes

Please rate the outcomes below on relevance and feasibility in the context of assessing the surgical learning curve of the neurosurgeon.

(1/24) BLOOD LOSS

Blood loss refers to the total amount of blood lost during a surgical procedure, which can potentially impact patient outcomes and recovery.

(2/24) PROCEDURE DURATION

Procedure duration refers to the total time taken to complete a surgical procedure, from the initiation of anesthesia to the closure of the surgical site.

(3/24) INTRA-OPERATIVE COMPLICATIONS

Intra-operative complications are adverse events or issues that occur during a surgical procedure, which can affect the patient's safety and surgical outcomes. These complications can range from minor incidents to severe problems, such as excessive bleeding or damage to surrounding tissues, and may necessitate additional interventions.

B1. Do you find this outcome relevant in order to asses the surgical learning curve of neurosurgeon operating on high-grade glioma?

Certainly not relevant 1	2	3	4	5	6	7	8	Very relevant 9
☐	☐	☐	☐	☐	☐	☐	☐	☐

B2. Do you consider it feasible to consistently measure this outcome in daily practice in the context of assessing a surgical learning curve?

Certainly not feasible 1	2	3	4	5	6	7	8	Very feasible 9
☐	☐	☐	☐	☐	☐	☐	☐	☐

B3. Do you find this outcome relevant in order to asses the surgical learning curve of neurosurgeon operating on high-grade glioma?

Certainly not relevant 1	2	3	4	5	6	7	8	Very relevant 9
☐	☐	☐	☐	☐	☐	☐	☐	☐

B4. Do you consider it feasible to consistently measure this outcome in daily practice in the context of assessing a surgical learning curve?

Certainly not feasible 1	2	3	4	5	6	7	8	Very feasible 9
☐	☐	☐	☐	☐	☐	☐	☐	☐

B5. Do you find this outcome relevant in order to asses the surgical learning curve of neurosurgeon operating on high-grade glioma?

Certainly not relevant 1	2	3	4	5	6	7	8	Very relevant 9
☐	☐	☐	☐	☐	☐	☐	☐	☐

B6. Do you consider it feasible to consistently measure this outcome in daily practice in the context of assessing a surgical learning curve?

Certainly not feasible 1	2	3	4	5	6	7	8	Very feasible 9
☐	☐	☐	☐	☐	☐	☐	☐	☐

B7. Comments

Please indicate here if you have any questions or comments (for example, regarding the wording of an outcome or if you would like to see something different/more specific) about any of the above outcomes. At the end of this questionnaire, you will have the opportunity to suggest additional outcomes that should be considered in this Delphi study, so you do not need to provide them here yet.

Section C: 1. OPERATIVE OUTCOMES: Surgeon level outcomes

Please rate the outcomes below on relevance and feasibility in the context of assessing the surgical learning curve of the neurosurgeon.

(4/24) EXECUTION OF THE INTENDED PLAN

Execution of the intended plan refers to the surgeon's ability to successfully carry out the preoperative surgical plan during the procedure. This outcome measure evaluates whether the surgery adhered to the intended course of action, including accuracy, technical success, efficiency and postoperative results.

(5/24) CONTROL OF INSTRUMENTS

Control of instruments refers to the surgeon's ability to effectively manipulate and operate surgical tools during a procedure.

(6/24) SURGEON FATIGUE

Surgeon fatigue refers to the physical and mental exhaustion experienced by surgeons due to the demanding nature of surgical procedures, long hours, and high-stress environments.

C1. Do you find this outcome relevant in order to asses the surgical learning curve of neurosurgeon operating on high-grade glioma?

Certainly not relevant 1	2	3	4	5	6	7	8	Very relevant 9
☐	☐	☐	☐	☐	☐	☐	☐	☐

C2. Do you consider it feasible to consistently measure this outcome in daily practice in the context of assessing a surgical learning curve?

Certainly not feasible 1	2	3	4	5	6	7	8	Very feasible 9
☐	☐	☐	☐	☐	☐	☐	☐	☐

C3. Do you find this outcome relevant in order to asses the surgical learning curve of neurosurgeon operating on high-grade glioma?

Certainly not relevant 1	2	3	4	5	6	7	8	Very relevant 9
☐	☐	☐	☐	☐	☐	☐	☐	☐

C4. Do you consider it feasible to consistently measure this outcome in daily practice in the context of assessing a surgical learning curve?

Certainly not feasible 1	2	3	4	5	6	7	8	Very feasible 9
☐	☐	☐	☐	☐	☐	☐	☐	☐

C5. Do you find this outcome relevant in order to asses the surgical learning curve of neurosurgeon operating on high-grade glioma?

Certainly not relevant 1	2	3	4	5	6	7	8	Very relevant 9
☐	☐	☐	☐	☐	☐	☐	☐	☐

C6. **Do you consider it feasible to consistently measure this outcome in daily practice in the context of assessing a surgical learning curve?**

Certainly not feasible 1	2	3	4	5	6	7	8	Very feasible 9
☐	☐	☐	☐	☐	☐	☐	☐	☐

C7. **Comments**

Please indicate here if you have any questions or comments (for example, regarding the wording of an outcome or if you would like to see something different/more specific) about any of the above outcomes. At the end of this questionnaire, you will have the opportunity to suggest additional outcomes that should be considered in this Delphi study, so you do not need to provide them here yet.

Section D: 1. OPERATIVE OUTCOMES: Organization level outcomes

Please rate the outcomes below on relevance and feasibility in the context of assessing the surgical learning curve of the neurosurgeon.

(7/24) STANDARDISATION OF OPERATIVE QUALITY

Standardization of surgical quality means that a neurosurgeon is able to create a workflow and approach for themselves, allowing them to perform each surgery in a standardized way and, in doing so, strive to ensure quality.

D1. **Do you find this outcome relevant in order to asses the surgical learning curve of neurosurgeon operating on high-grade glioma?**

Certainly not relevant 1	2	3	4	5	6	7	8	Very relevant 9
☐	☐	☐	☐	☐	☐	☐	☐	☐

D2. **Do you consider it feasible to consistently measure this outcome in daily practice in the context of assessing a surgical learning curve?**

Certainly not feasible 1	2	3	4	5	6	7	8	Very feasible 9
☐	☐	☐	☐	☐	☐	☐	☐	☐

D3. **Comments**

Please indicate here if you have any questions or comments (for example, regarding the wording of an outcome or if you would like to see something different/more specific) about any of the above outcomes. At the end of this questionnaire, you will have the opportunity to suggest additional outcomes that should be considered in this Delphi study, so you do not need to provide them here yet.

Section E: 2. PATIENT OUTCOMES: General patient outcomes

Please rate the outcomes below on relevance and feasibility in the context of assessing the surgical learning curve of the neurosurgeon.

(8/24) PERCENTAGE OF TUMOR RESECTED

Percentage of tumor resected refers to the proportion of the tumor that is successfully removed during the surgical procedure compared to its total volume.

(9/24) RESIDUAL TUMOR REMNANT

Residual tumor volume refers to the amount of tumor tissue that remains in the brain after the surgical resection.

(10/24) LENGTH OF HOSPITAL STAY

Length of hospital stay refers to the total time a patient spends in the hospital following a surgical procedure.

(11/24) LENGTH OF ICU/BRAIN CARE UNIT/RECOVERY STAY

Length of ICU/brain care unit/recovery stay (depending on local work flows) refers to the total time a patient spends in this unit following the surgical procedure or medical intervention.

(12/24) READMISSION RATE

E1. **Do you find this outcome relevant in order to asses the surgical learning curve of neurosurgeon operating on high-grade glioma?**

Certainly not relevant 1	2	3	4	5	6	7	8	Very relevant 9
☐	☐	☐	☐	☐	☐	☐	☐	☐

E2. **Do you consider it feasible to consistently measure this outcome in daily practice in the context of assessing a surgical learning curve?**

Certainly not feasible 1	2	3	4	5	6	7	8	Very feasible 9
☐	☐	☐	☐	☐	☐	☐	☐	☐

LimeSurvey

E3. Do you find this outcome relevant in order to asses the surgical learning curve of neurosurgeon operating on high-grade glioma?

Certainly not relevant 1	2	3	4	5	6	7	8	Very relevant 9
☐	☐	☐	☐	☐	☐	☐	☐	☐

E4. Do you consider it feasible to consistently measure this outcome in daily practice in the context of assessing a surgical learning curve?

Certainly not feasible 1	2	3	4	5	6	7	8	Very feasible 9
☐	☐	☐	☐	☐	☐	☐	☐	☐

E5. Do you find this outcome relevant in order to asses the surgical learning curve of neurosurgeon operating on high-grade glioma?

Certainly not relevant 1	2	3	4	5	6	7	8	Very relevant 9
☐	☐	☐	☐	☐	☐	☐	☐	☐

E6. Do you consider it feasible to consistently measure this outcome in daily practice in the context of assessing a surgical learning curve?

Certainly not feasible 1	2	3	4	5	6	7	8	Very feasible 9
☐	☐	☐	☐	☐	☐	☐	☐	☐

E7. Do you find this outcome relevant in order to asses the surgical learning curve of neurosurgeon operating on high-grade glioma?

Certainly not relevant 1	2	3	4	5	6	7	8	Very relevant 9
☐	☐	☐	☐	☐	☐	☐	☐	☐

E8. Do you consider it feasible to consistently measure this outcome in daily practice in the context of assessing a surgical learning curve?

Certainly not feasible 1	2	3	4	5	6	7	8	Very feasible 9
☐	☐	☐	☐	☐	☐	☐	☐	☐

E9. Do you find this outcome relevant in order to asses the surgical learning curve of neurosurgeon operating on high-grade glioma?

Certainly not relevant 1	2	3	4	5	6	7	8	Very relevant 9
☐	☐	☐	☐	☐	☐	☐	☐	☐

LimeSurvey

E10. Do you consider it feasible to consistently measure this outcome in daily practice in the context of assessing a surgical learning curve?

Certainly not feasible 1	2	3	4	5	6	7	8	Very feasible 9
☐	☐	☐	☐	☐	☐	☐	☐	☐

E11. Do you find this outcome relevant in order to asses the surgical learning curve of neurosurgeon operating on high-grade glioma?

Certainly not relevant 1	2	3	4	5	6	7	8	Very relevant 9
☐	☐	☐	☐	☐	☐	☐	☐	☐

E12. Do you consider it feasible to consistently measure this outcome in daily practice in the context of assessing a surgical learning curve?

Certainly not feasible 1	2	3	4	5	6	7	8	Very feasible 9
☐	☐	☐	☐	☐	☐	☐	☐	☐

E13. Do you find this outcome relevant in order to asses the surgical learning curve of neurosurgeon operating on high-grade glioma?

Certainly not relevant 1	2	3	4	5	6	7	8	Very relevant 9
☐	☐	☐	☐	☐	☐	☐	☐	☐

E14. Do you consider it feasible to consistently measure this outcome in daily practice in the context of assessing a surgical learning curve?

Certainly not feasible 1	2	3	4	5	6	7	8	Very feasible 9
☐	☐	☐	☐	☐	☐	☐	☐	☐

E15. Comments

Please indicate here if you have any questions or comments (for example, regarding the wording of an outcome or if you would like to see something different/more specific) about any of the above outcomes. At the end of this questionnaire, you will have the opportunity to suggest additional outcomes that should be considered in this Delphi study, so you do not need to provide them here yet.

Section F: 2. PATIENT OUTCOMES: Functional ability/impairment

Please rate the outcomes below on relevance and feasibility in the context of assessing the surgical learning curve of the neurosurgeon.

(15/24) TRANSIENT POST-OPERATIVE NEUROLOGICAL SYMPTOMS OR DETERIORATION

Transient post-operative neurological symptoms or deterioration refers to the occurrence of new or worsening neurological deficits which resolve within 30 days after the surgical procedure.

(16/24) PERMANENT POST-OPERATIVE NEUROLOGICAL SYMPTOMS OR DETERIORATION

Permanent post-operative neurological symptoms or deterioration refers to the occurrence of new or worsening neurological deficits which do <u>not resolve</u> within 30 days following the surgical procedure.

F1. Do you find this outcome relevant in order to asses the surgical learning curve of neurosurgeon operating on high-grade glioma?

Certainly not relevant 1	2	3	4	5	6	7	8	Very relevant 9
☐	☐	☐	☐	☐	☐	☐	☐	☐

F2. Do you consider it feasible to consistently measure this outcome in daily practice in the context of assessing a surgical learning curve?

Certainly not feasible 1	2	3	4	5	6	7	8	Very feasible 9
☐	☐	☐	☐	☐	☐	☐	☐	☐

F3. Do you find this outcome relevant in order to asses the surgical learning curve of neurosurgeon operating on high-grade glioma?

Certainly not relevant 1	2	3	4	5	6	7	8	Very relevant 9
☐	☐	☐	☐	☐	☐	☐	☐	☐

F4. Do you consider it feasible to consistently measure this outcome in daily practice in the context of assessing a surgical learning curve?

Certainly not feasible 1	2	3	4	5	6	7	8	Very feasible 9
☐	☐	☐	☐	☐	☐	☐	☐	☐

F5. Comments

Please indicate here if you have any questions or comments (for example, regarding the wording of an outcome or if you would like to see something different/more specific) about any of the above outcomes. At the end of this questionnaire, you will have the opportunity to suggest additional outcomes that should be considered in this Delphi study, so you do not need to provide them here yet.

Section G: 2. PATIENT OUTCOMES: Adverse events

Please rate the outcomes below on relevance and feasibility in the context of assessing the surgical learning curve of the neurosurgeon.

(17/24) ALL ADVERSE EVENTS

G1. Do you find this outcome relevant in order to asses the surgical learning curve of neurosurgeon operating on high-grade glioma?

Certainly not relevant 1	2	3	4	5	6	7	8	Very relevant 9
☐	☐	☐	☐	☐	☐	☐	☐	☐

G2. Do you consider it feasible to consistently measure this outcome in daily practice in the context of assessing a surgical learning curve?

Certainly not feasible 1	2	3	4	5	6	7	8	Very feasible 9
☐	☐	☐	☐	☐	☐	☐	☐	☐

G3. Do you find this outcome relevant in order to asses the surgical learning curve of neurosurgeon operating on high-grade glioma?

Certainly not relevant 1	2	3	4	5	6	7	8	Very relevant 9
☐	☐	☐	☐	☐	☐	☐	☐	☐

G4. Do you consider it feasible to consistently measure this outcome in daily practice in the context of assessing a surgical learning curve?

Certainly not feasible 1	2	3	4	5	6	7	8	Very feasible 9
☐	☐	☐	☐	☐	☐	☐	☐	☐

LimeSurvey

G5. **Do you find this outcome relevant in order to asses the surgical learning curve of neurosurgeon operating on high-grade glioma?**

Certainly not relevant 1	2	3	4	5	6	7	8	Very relevant 9
☐	☐	☐	☐	☐	☐	☐	☐	☐

G6. **Do you consider it feasible to consistently measure this outcome in daily practice in the context of assessing a surgical learning curve?**

Certainly not feasible 1	2	3	4	5	6	7	8	Very feasible 9
☐	☐	☐	☐	☐	☐	☐	☐	☐

G7. **Do you find this outcome relevant in order to asses the surgical learning curve of neurosurgeon operating on high-grade glioma?**

Certainly not relevant 1	2	3	4	5	6	7	8	Very relevant 9
☐	☐	☐	☐	☐	☐	☐	☐	☐

G8. **Do you consider it feasible to consistently measure this outcome in daily practice in the context of assessing a surgical learning curve?**

Certainly not feasible 1	2	3	4	5	6	7	8	Very feasible 9
☐	☐	☐	☐	☐	☐	☐	☐	☐

G9. **Comments**

Please indicate here if you have any questions or comments (for example, regarding the wording of an outcome or if you would like to see something different/more specific) about any of the above outcomes. At the end of this questionnaire, you will have the opportunity to suggest additional outcomes that should be considered in this Delphi study, so you do not need to provide them here yet.

Section H: 2. PATIENT OUTCOMES: Mortality

Please rate the outcomes below on relevance and feasibility in the context of assessing the surgical learning curve of the neurosurgeon.

(21/24) MORTALITY RATE

The mortality rate is a measure of the frequency of occurrence of death in a defined population during a specified period. This outcome refers, in this context, to the overall mortality rate.

(22/24) MORTALITY RATE

H1. Do you find this outcome relevant in order to assess the surgical learning curve of neurosurgeon operating on high-grade glioma?

Certainly not relevant 1	2	3	4	5	6	7	8	Very relevant 9
☐	☐	☐	☐	☐	☐	☐	☐	☐

H2. Do you consider it feasible to consistently measure this outcome in daily practice in the context of assessing a surgical learning curve?

Certainly not feasible 1	2	3	4	5	6	7	8	Very feasible 9
☐	☐	☐	☐	☐	☐	☐	☐	☐

H3. Do you find this outcome relevant in order to assess the surgical learning curve of neurosurgeon operating on high-grade glioma?

Certainly not relevant 1	2	3	4	5	6	7	8	Very relevant 9
☐	☐	☐	☐	☐	☐	☐	☐	☐

H4. Do you consider it feasible to consistently measure this outcome in daily practice in the context of assessing a surgical learning curve?

Certainly not feasible 1	2	3	4	5	6	7	8	Very feasible 9
☐	☐	☐	☐	☐	☐	☐	☐	☐

H5. Comments

Please indicate here if you have any questions or comments (for example, regarding the wording of an outcome or if you would like to see something different/more specific) about any of the above outcomes. At the end of this questionnaire, you will have the opportunity to suggest additional outcomes that should be considered in this Delphi study, so you do not need to provide them here yet.

Section I: 3. PATIENT-REPORTED OUTCOMES

Please rate the outcomes below on relevance and feasibility in the context of assessing the surgical learning curve of the neurosurgeon.

(23/24) DISEASE RELATED QUALITY OF LIFE 30 DAYS AFTER SURGERY

Disease-related quality of life one month after surgery can be measured using disease-specific quality of life questionnaires (for example EORTC QLQ-C30/BN20) designed to assess the impact of the disease and treatment on the patient's well-being and daily functioning.

(24/24) OVERALL QUALITY OF LIFE 30 DAYS AFTER SURGERY

Overall quality of life one month after surgery can be measured using general quality of life questionnaires (for example EQ5D-5L) designed to assess an individual's general well-being and life satisfaction, encompassing physical, mental, and social health dimensions.

I1. Do you find this outcome relevant in order to assess the surgical learning curve of neurosurgeon operating on high-grade glioma?

Certainly not relevant 1	2	3	4	5	6	7	8	Very relevant 9
☐	☐	☐	☐	☐	☐	☐	☐	☐

I2. Do you consider it feasible to consistently measure this outcome in daily practice in the context of assessing a surgical learning curve?

Certainly not feasible 1	2	3	4	5	6	7	8	Very feasible 9
☐	☐	☐	☐	☐	☐	☐	☐	☐

I3. Do you find this outcome relevant in order to assess the surgical learning curve of neurosurgeon operating on high-grade glioma?

Certainly not relevant 1	2	3	4	5	6	7	8	Very relevant 9
☐	☐	☐	☐	☐	☐	☐	☐	☐

I4. Do you consider it feasible to consistently measure this outcome in daily practice in the context of assessing a surgical learning curve?

Certainly not feasible 1	2	3	4	5	6	7	8	Very feasible 9
☐	☐	☐	☐	☐	☐	☐	☐	☐

LimeSurvey

I5. **Comments**

Please indicate here if you have any questions or comments (for example, regarding the wording of an outcome or if you would like to see something different/more specific) about any of the above outcomes. At the end of this questionnaire, you will have the opportunity to suggest additional outcomes that should be considered in this Delphi study, so you do not need to provide them here yet.

LimeSurvey

Section J: TOP 5 AND SUGGESTION FOR OTHER OUTCOMES

J1. TOP 5

Choose the <u>5 outcomes</u> from the list below that you consider to be the most important in the context of assessing the surgical learning curve of a neurosurgeon when operating on high-grade gliomas.

Blood loss ☐☐
Procedure duration ☐☐
Intra-operative complications ☐☐
Execution of the intended plan ☐☐
Control of instruments ☐☐
Surgeon fatigue ☐☐
Standardization of operative quality ☐☐
Percentage of tumor resected ☐☐
Residual tumor remnant ☐☐
Length of hospital stay ☐☐
Length of ICU/brain care unit/recovery stay ☐☐
Readmission rate <30 days ☐☐
Reoperation rate <30 days ☐☐
Initiation of adjuvant therapy <6 weeks ☐☐
Transient post-operative neurological symptoms or detoration ☐☐
Permanent post-operative neurological symptoms or detoration ☐☐
All adverse events <72 hours ☐☐
All adverse events <30 days ☐☐
Adverse events CD ≥ 2 <72 hours ☐☐
Adverse events CD ≥ 2 <30 days ☐☐
Mortality rate ☐☐
Mortality rate <30 days ☐☐
Disease related quality of life 30 days after surgery ☐☐
Overall quality of life 30 days after surgery ☐☐

J2. **SUGGESTIONS FOR OTHER OUTCOMES**

If you have suggestions for outcomes that are not mentioned in this questionnaire but you believe are important for assessing the surgical learning curve of a neurosurgeon and should be considered in the next Delphi round, please list them below.

Section K: CLOSING QUESTIONS

K1. **Online expert meeting**

Would you be interested in participating in the single-session online expert meeting of this Delphi study? This meeting constitutes the final round of the Delphi study, during which the definitive list of outcomes will be established. The online meeting will likely take place in the spring of 2025, and we expect it to last approximately 1 to 1.5 hours.

Yes ☐

No ☐

K2. **If you indicated interest in participating in the expert meeting, we kindly ask you to provide your email address below.**

K3. **General Comments**

Please use the space below to share any remarks about the surveyed outcomes or about this Delphi questionnaire overall.

You have reached the end of the questionnaire. Thank you for your participation in this Delphi study.
