# Supplementary material for: Core outcomes for assessing surgical learning curves in high-grade glioma surgery: a European Delphi study
Source: Brain Spine. 2026 May 16;6:106097. doi: 10.1016/j.bas.2026.106097 (PMC13397579; doi:10.1016/j.bas.2026.106097)
Supplement: Multimedia component 2 [file mmc2.pdf]

Supplementary Item 2

Core outcomes for assessing surgical learning curves in high-grade glioma surgery: a European Delphi study

Céline L.G. Neutel, MD[1], Valerie Diederen[1], Jiri Bartek, MD, PhD[2], Gerjon Hannink, PhD[3], Maroeska M. Rovers, PhD[3], Mark ter Laan, MD, PhD[1], the Expert Meeting Group[#]

[1] Department of Neurosurgery, Radboud university medical center, Nijmegen, The Netherlands.

[2] Department of Neurosurgery and Clinical Neuroscience, Karolinska University Hospital and Karolinska Institutet, Stockholm, Sweden

[3] Department of Medical Imaging, Radboud university medical center, Nijmegen, The Netherlands.

#

- Johnny Duerinck, MD, PhD, Department of Neurosurgery, Universitair Ziekenhuis Brussel, Vrije Universiteit Brussel, Brussels, Belgium
- Steven De Vleeschouwer, MD, PhD, Department of Neurosurgery, University Hospitals Leuven, Belgium and Department of Neurosciences, Leuven Brain Institute, KU Leuven, Belgium
- Tomas Kazda, MD, PhD, Department of Radiation oncology, Masaryk Memorial Cancer Institute, Brno, Czech Republic
- Alessia Pellerino, MD, PhD, Department of Neuroscience "Rita Levi Montalcini", University and City of Health and Science Hospital, Turin, Italy
- Michael Veldeman MD PhD, Department of Neurosurgery, RWTH Aachen University Hospital, Aachen, Germany
- Asgeir S. Jakola, MD, PhD, Institute of Neuroscience and Physiology, Department of Clinical Neuroscience, University of Gothenburg, Gothenburg, Sweden and Region Västra Götaland, Sahlgrenska University Hospital, Department of Neurosurgery, Gothenburg, Sweden
- Kostas N. Fountas, MD, PhD, Department of Neurosurgery, Faculty of Medicine, School of Health Sciences, University of Thessaly, Larisa, Greece
- Sebastian Pavel, MD, Brain Institute, Monza Hospital, Bucharest, Romania
- Dan-Andrei Mitrea, MD, Neuroaxis - Neurology Clinic, Bucharest, Romania

Supplementary Item 2

Second digital questionnaire

Dear respondent,

Welcome to the second and final questionnaire of the Delphi study 'Obtaining consensus for assessing surgical learning curves in high-grade glioma surgery: a European Delphi study.' Participation in the first questionnaire is not a requirement to complete this questionnaire.

The aim of this Delphi study is to achieve consensus on which outcomes we should consider and include for assessing the surgical learning curve of a neurosurgeon when operating on high-grade gliomas. This is important because neurosurgery is a rapidly evolving field with innovations emerging at a fast pace. Measuring the surgical learning curve can be valuable in clinical practice, in the training of neurosurgeons, and within medical research for evaluating new interventions.

Given the uncertainty regarding important outcome measures for assessing this learning curve, which may differ from those used to evaluate treatment effectiveness, we have initiated this study. A Delphi study consists of several steps, and this questionnaire is the second step. We hope to gather input from as many stakeholders in neuro-oncology as possible regarding which outcomes they believe are important.

Section A: INTRODUCTION AND DEMOGRAPHICS

This Delphi study aims to identify the most relevant outcomes for assessing the learning curve of neurosurgeons. The goal is to determine which aspects of surgical skills, operative organization, and patient outcomes provide the best insight into a neurosurgeon's professional development over time. This is the second and final questionnaire for this Delphi study. Participation in the first round is not required to complete this second questionnaire.

Two outcomes from the first questionnaire (namely the percentage tumor resected and the residual tumor remnant) have been accepted as relevant and feasible, and will therefore not be reassessed. In this questionnaire, some outcomes will be re-evaluated for both relevance and feasibility (18 outcomes). Other outcomes will be tested for either relevance (4 outcomes) or feasibility (5 outcomes).

For the outcomes being revisited, we have clarified or added details where needed and included the medians and IQRs from the first questionnaire. These scores provide you with insight into the opinions and ideas of the respondents from the first questionnaire and may help you in scoring the outcomes of this questionnaire. We have also included new outcomes suggested by respondents in the first questionnaire.

For respondents who completed the first questionnaire: you will notice that some questions need to be answered again. This is part of the Delphi methodology. Additionally, since the questionnaires are anonymous, we kindly ask you to provide your demographic information once more. This is essential for the reporting of the study results.

We estimate that completing this questionnaire will require approximately 10 to 15 minutes.

It is possible to go back to a previous question. To do so, use the "Previous" button at the bottom left of the page, not the "Back" button on your internet browser.

If you have any questions before, during, or after completing the questionnaire, please feel free to contact the research team at celine.neutel@radboudumc.nl

----

Additional information:

The outcomes in this questionnaire are grouped as "outcomes to be tested for both relevance and feasibility," "outcomes to be tested only for relevance," and "outcomes to be tested only for feasibility." This has been done to avoid confusion when scoring.

At the end of the questionnaire, you will be asked to compile a top 5 list of the outcomes you consider most important for assessing a neurosurgeon's learning curve.There will also be an opportunity for you to suggest additional outcomes that you believe should be considered as well.

A1. What is your age?

Younger than 25 years ☐

25 - 34 years ☐

35 - 44 years ☐

45 - 54 years ☐

A2. In which country do you currently work?

- Albania ☐
- Andorra ☐
- Austria ☐
- Belgium ☐
- Bosnia and Herzegovina ☐
- Bulgaria ☐
- Croatia ☐
- Cyprus ☐
- Czech Republic ☐
- Denmark ☐
- Estonia ☐
- Finland ☐
- France ☐
- Greece ☐
- Germany ☐
- Hungary ☐
- Ireland ☐
- Iceland ☐
- Italy ☐
- Kosovo ☐
- Latvia ☐
- Liechtenstein ☐
- Lithuania ☐
- Luxembourg ☐
- Malta ☐
- Moldova ☐
- Monaco ☐
- Montenegro ☐
- North Macedonia ☐
- Norway ☐
- Poland ☐

A3. **What is your current profession?**

- Neurosurgeon ☐
- Neurologist / Neuro-oncologist ☐
- Radiologist ☐
- Radiotherapist ☐
- Specialized nurse ☐
- Other ☐

Other

A4. **Are you working in an academic or non-academic hospital?**

- Academic hospital ☐
- Non-academic hospital ☐

A5. **Approximately how many high-grade gliomas are operated on annually at the center where you work?**

- < 10 ☐
- 10 - 50 ☐
- 51 - 150 ☐
- > 150 ☐
- I don't know ☐

A6. **Do you hold a position in a national working group involved in developing guidelines and/or creating policies related to neuro-oncological care?**

- Yes ☐
- No ☐

A7. How many years of experience do you have in neuro-oncological care?

Please exclude your medical specialist training, and include any fellowships.

- 0 - 5 years ☐
- 6 - 10 years ☐
- 11 - 15 years ☐
- 16 years or more ☐
- Not specialized in neuro-oncology ☐

A8. If you are open to any follow-up questions regarding your answers, please leave your email address below.

Section B: 1. Outcomes tested for RELEVANCE and FEASIBILITY

Please rate the outcomes below on relevance and feasibility in the context of assessing the surgical learning curve of the neurosurgeon.

(1/27) BLOOD LOSS

Blood loss refers to the total amount of blood lost during a surgical procedure, which can potentially impact patient outcomes and recovery.

(2/27) PROCEDURE DURATION

Procedure duration refers to the total time taken to complete a surgical procedure, from the initiation of anesthesia to the closure of the surgical site.

(3/27) SURGEON FATIGUE

Surgeon fatigue refers to the physical and mental exhaustion experienced by surgeons due to the demanding nature of surgical procedures, long hours, and high-stress environments. This could be measured by using AI-driven technologies, wearable devices and real-time feedback systems. Techniques such as heart rate variability analysis, electroencephalogram monitoring, and computer vision-based behavioural analysis could be used.

(4/27) STANDARDISATION OF OPERATIVE WORKFLOW

Standardization of operative workflow means that a neurosurgeon is able to create a workflow and approach for themselves, allowing them to perform each surgery in a standardized way and, in doing so, strive to ensure quality. This could be measured for example with machine learning-based surgical workflow analysis (PMID: 34740198) or by supervisor measurements and assessment (e.g. repositioning of instruments, additional hemostasis).

(5/27) LENGTH OF ICU/BRAIN CARE UNIT/RECOVERY STAY

Length of ICU/brain care unit/recovery stay (depending on local work flows) refers to the total time a patient spends in this unit following the surgical procedure.

(6/27) LENGTH OF HOSPITAL STAY

Length of hospital stay refers to the total time a patient spends in the hospital following a surgical procedure.

(7/27) INITIATION OF ADJUVANT THERAPY

B1. Do you find this outcome relevant in order to asses the surgical learning curve of neurosurgeon operating on high-grade glioma?

Median (IQR) score first questionnaire: 6 (4-7)

Certainly not relevant 1	2	3	4	5	6	7	8	Very relevant ? 9
☐	☐	☐	☐	☐	☐	☐	☐	☐

LimeSurvey

B2. **Do you consider it feasible to consistently measure this outcome in daily practice in the context of assessing a surgical learning curve?**

Median (IQR) score first questionnaire: 7,5 (5-8)

Certainly not feasible 1	2	3	4	5	6	7	8	Very feasible 9
☐	☐	☐	☐	☐	☐	☐	☐	☐

B3. **Do you find this outcome relevant in order to asses the surgical learning curve of neurosurgeon operating on high-grade glioma?**

Median score (IQR) first questionnaire: 6 (5-7)

Certainly not relevant 1	2	3	4	5	6	7	8	Very relevant 9
☐	☐	☐	☐	☐	☐	☐	☐	☐

B4. **Do you consider it feasible to consistently measure this outcome in daily practice in the context of assessing a surgical learning curve?**

Median score (IQR) first questionnaire: 8 (6.25-9)

Certainly not feasible 1	2	3	4	5	6	7	8	Very feasible 9
☐	☐	☐	☐	☐	☐	☐	☐	☐

B5. **Do you find this outcome relevant in order to asses the surgical learning curve of neurosurgeon operating on high-grade glioma?**

Median score (IQR) first questionnaire: 6 (4-7)

Certainly not relevant 1	2	3	4	5	6	7	8	Very relevant 9
☐	☐	☐	☐	☐	☐	☐	☐	☐

B6. **Do you consider it feasible to consistently measure this outcome in daily practice in the context of assessing a surgical learning curve?**

Median score (IQR) first questionnaire: 4 (3-6)

Certainly not feasible 1	2	3	4	5	6	7	8	Very feasible 9
☐	☐	☐	☐	☐	☐	☐	☐	☐

B7. **Do you find this outcome relevant in order to asses the surgical learning curve of neurosurgeon operating on high-grade glioma?**

Median score (IQR) first questionnaire: 7,5 (6-8)

Certainly not relevant 1	2	3	4	5	6	7	8	Very relevant 9
☐	☐	☐	☐	☐	☐	☐	☐	☐

B8. **Do you consider it feasible to consistently measure this outcome in daily practice in the context of assessing a surgical learning curve?**

Median score (IQR) first questionnaire: 6 (4.25-7.75)

Certainly not feasible 1	2	3	4	5	6	7	8	Very feasible 9
☐	☐	☐	☐	☐	☐	☐	☐	☐

B9. **Do you find this outcome relevant in order to asses the surgical learning curve of neurosurgeon operating on high-grade glioma?**

Median score (IQR) first questionnaire: 6 (4-7)

Certainly not relevant 1	2	3	4	5	6	7	8	Very relevant 9
☐	☐	☐	☐	☐	☐	☐	☐	☐

B10. **Do you consider it feasible to consistently measure this outcome in daily practice in the context of assessing a surgical learning curve?**

Median score (IQR) first questionnaire: 8 (6-9)

Certainly not feasible 1	2	3	4	5	6	7	8	Very feasible 9
☐	☐	☐	☐	☐	☐	☐	☐	☐

B11. **Do you find this outcome relevant in order to asses the surgical learning curve of neurosurgeon operating on high-grade glioma?**

Median score (IQR) first questionnaire: 6 (4-6.75)

Certainly not relevant 1	2	3	4	5	6	7	8	Very relevant 9
☐	☐	☐	☐	☐	☐	☐	☐	☐

B12. **Do you consider it feasible to consistently measure this outcome in daily practice in the context of assessing a surgical learning curve?**

Median score (IQR) first questionnaire: 8 (6-9)

Certainly not feasible 1	2	3	4	5	6	7	8	Very feasible 9
☐	☐	☐	☐	☐	☐	☐	☐	☐

B13. **Do you find this outcome relevant in order to asses the surgical learning curve of neurosurgeon operating on high-grade glioma?**

Median score (IQR) first questionnaire: 5,5 (3-7)

Certainly not relevant 1	2	3	4	5	6	7	8	Very relevant 9
☐	☐	☐	☐	☐	☐	☐	☐	☐

LimeSurvey

B14. **Do you consider it feasible to consistently measure this outcome in daily practice in the context of assessing a surgical learning curve?**

Median score (IQR) first questionnaire: 8 (6.25-9)

Certainly not feasible 1	2	3	4	5	6	7	8	Very feasible 9
☐	☐	☐	☐	☐	☐	☐	☐	☐

B15. **Do you find this outcome relevant in order to asses the surgical learning curve of neurosurgeon operating on high-grade glioma?**

Median score (IQR) first questionnaire: 7 (4.25-8)

Certainly not relevant 1	2	3	4	5	6	7	8	Very relevant 9
☐	☐	☐	☐	☐	☐	☐	☐	☐

B16. **Do you consider it feasible to consistently measure this outcome in daily practice in the context of assessing a surgical learning curve?**

Median score (IQR) first questionnaire: 7 (6-8)

Certainly not feasible 1	2	3	4	5	6	7	8	Very feasible 9
☐	☐	☐	☐	☐	☐	☐	☐	☐

B17. **Do you find this outcome relevant in order to asses the surgical learning curve of neurosurgeon operating on high-grade glioma?**

Median score (IQR) first questionnaire: 7 (6-8)

Certainly not relevant 1	2	3	4	5	6	7	8	Very relevant 9
☐	☐	☐	☐	☐	☐	☐	☐	☐

B18. **Do you consider it feasible to consistently measure this outcome in daily practice in the context of assessing a surgical learning curve?**

Median score (IQR) first questionnaire: 7 (6-8)

Certainly not feasible 1	2	3	4	5	6	7	8	Very feasible 9
☐	☐	☐	☐	☐	☐	☐	☐	☐

B19. **Do you find this outcome relevant in order to asses the surgical learning curve of neurosurgeon operating on high-grade glioma?**

Median score (IQR) first questionnaire: 6 (5.25-7)

Certainly not relevant 1	2	3	4	5	6	7	8	Very relevant 9
☐	☐	☐	☐	☐	☐	☐	☐	☐

LimeSurvey

B20. **Do you consider it feasible to consistently measure this outcome in daily practice in the context of assessing a surgical learning curve?**

Median score (IQR) first questionnaire: 7 (5-8)

Certainly not feasible 1	2	3	4	5	6	7	8	Very feasible 9
☐	☐	☐	☐	☐	☐	☐	☐	☐

B21. **Do you find this outcome relevant in order to asses the surgical learning curve of neurosurgeon operating on high-grade glioma?**

Median score (IQR) first questionnaire: 7 (6-8)

Certainly not relevant 1	2	3	4	5	6	7	8	Very relevant 9
☐	☐	☐	☐	☐	☐	☐	☐	☐

B22. **Do you consider it feasible to consistently measure this outcome in daily practice in the context of assessing a surgical learning curve?**

Median score (IQR) first questionnaire: 7 (6-8)

Certainly not feasible 1	2	3	4	5	6	7	8	Very feasible 9
☐	☐	☐	☐	☐	☐	☐	☐	☐

B23. **Do you find this outcome relevant in order to assess the surgical learning curve of neurosurgeon operating on high-grade glioma?**

Median score (IQR) first questionnaire: 7 (6-8)

Certainly not relevant 1	2	3	4	5	6	7	8	Very relevant 9
☐	☐	☐	☐	☐	☐	☐	☐	☐

B24. **Do you consider it feasible to consistently measure this outcome in daily practice in the context of assessing a surgical learning curve?**

Median score (IQR) first questionnaire: 6 (5-8)

Certainly not feasible 1	2	3	4	5	6	7	8	Very feasible 9
☐	☐	☐	☐	☐	☐	☐	☐	☐

B25. **Do you find this outcome relevant in order to assess the surgical learning curve of neurosurgeon operating on high-grade glioma?**

Median score (IQR) first questionnaire: 7 (6-8)

Certainly not relevant 1	2	3	4	5	6	7	8	Very relevant 9
☐	☐	☐	☐	☐	☐	☐	☐	☐

LimeSurvey

B26. **Do you consider it feasible to consistently measure this outcome in daily practice in the context of assessing a surgical learning curve?**

Median score (IQR) first questionnaire: 6,5 (5-8)

Certainly not feasible 1	2	3	4	5	6	7	8	Very feasible 9
☐	☐	☐	☐	☐	☐	☐	☐	☐

B27. **Do you find this outcome relevant in order to assess the surgical learning curve of neurosurgeon operating on high-grade glioma?**

Certainly not relevant 1	2	3	4	5	6	7	8	Very relevant 9
☐	☐	☐	☐	☐	☐	☐	☐	☐

B28. **Do you consider it feasible to consistently measure this outcome in daily practice in the context of assessing a surgical learning curve?**

Certainly not feasible 1	2	3	4	5	6	7	8	Very feasible 9
☐	☐	☐	☐	☐	☐	☐	☐	☐

B29. **Do you find this outcome relevant in order to assess the surgical learning curve of neurosurgeon operating on high-grade glioma?**

Certainly not relevant 1	2	3	4	5	6	7	8	Very relevant 9
☐	☐	☐	☐	☐	☐	☐	☐	☐

B30. **Do you consider it feasible to consistently measure this outcome in daily practice in the context of assessing a surgical learning curve?**

Certainly not feasible 1	2	3	4	5	6	7	8	Very feasible 9
☐	☐	☐	☐	☐	☐	☐	☐	☐

B31. **Do you find this outcome relevant in order to assess the surgical learning curve of neurosurgeon operating on high-grade glioma?**

Certainly not relevant 1	2	3	4	5	6	7	8	Very relevant 9
☐	☐	☐	☐	☐	☐	☐	☐	☐

B32. **Do you consider it feasible to consistently measure this outcome in daily practice in the context of assessing a surgical learning curve?**

Certainly not feasible 1	2	3	4	5	6	7	8	Very feasible 9
☐	☐	☐	☐	☐	☐	☐	☐	☐

B33. **Do you find this outcome relevant in order to assess the surgical learning curve of neurosurgeon operating on high-grade glioma?**

Certainly not relevant 1	2	3	4	5	6	7	8	Very relevant 9
☐	☐	☐	☐	☐	☐	☐	☐	☐

LimeSurvey

B34. **Do you consider it feasible to consistently measure this outcome in daily practice in the context of assessing a surgical learning curve?**

Certainly not feasible 1	2	3	4	5	6	7	8	Very feasible 9
☐	☐	☐	☐	☐	☐	☐	☐	☐

B35. **Do you find this outcome relevant in order to assess the surgical learning curve of neurosurgeon operating on high-grade glioma?**

Certainly not relevant 1	2	3	4	5	6	7	8	Very relevant 9
☐	☐	☐	☐	☐	☐	☐	☐	☐

B36. **Do you consider it feasible to consistently measure this outcome in daily practice in the context of assessing a surgical learning curve?**

Certainly not feasible 1	2	3	4	5	6	7	8	Very feasible 9
☐	☐	☐	☐	☐	☐	☐	☐	☐

B37. Comments

Please indicate here if you have any questions or comments (for example, regarding the wording of an outcome or if you would like to see something different/more specific) about any of the above outcomes. At the end of this questionnaire, you will have the opportunity to suggest additional outcomes that should be considered in this Delphi study, so you do not need to provide them here yet.

Section C: 2. Outcomes tested for RELEVANCE

Please rate the outcomes below on relevance in the context of assessing the surgical learning curve of the neurosurgeon.

All outcomes below scored with consensus as sufficient for feasibility in the first questionnaire round.

(19/27) READMISSION RATE

C1. **Do you find this outcome relevant in order to asses the surgical learning curve of neurosurgeon operating on high-grade glioma?**

Median score (IQR) first questionnaire: 7 (5.25-8)

Certainly not relevant 1	2	3	4	5	6	7	8	Very relevant 9
☐	☐	☐	☐	☐	☐	☐	☐	☐

C2. **Do you find this outcome relevant in order to asses the surgical learning curve of neurosurgeon operating on high-grade glioma?**

Median score (IQR) first questionnaire: 7 (6.25-8.75)

Certainly not relevant 1	2	3	4	5	6	7	8	Very relevant 9
☐	☐	☐	☐	☐	☐	☐	☐	☐

C3. **Do you find this outcome relevant in order to assess the surgical learning curve of neurosurgeon operating on high-grade glioma?**

Median score (IQR) first questionnaire: 6 (5-8)

Certainly not relevant 1	2	3	4	5	6	7	8	Very relevant 9
☐	☐	☐	☐	☐	☐	☐	☐	☐

C4. **Do you find this outcome relevant in order to assess the surgical learning curve of neurosurgeon operating on high-grade glioma?**

Median score (IQR) first questionnaire: 8 (6-9)

Certainly not relevant 1	2	3	4	5	6	7	8	Very relevant 9
☐	☐	☐	☐	☐	☐	☐	☐	☐

LimeSurvey

C5. Comments

Please indicate here if you have any questions or comments (for example, regarding the wording of an outcome or if you would like to see something different/more specific) about any of the above outcomes. At the end of this questionnaire, you will have the opportunity to suggest additional outcomes that should be considered in this Delphi study, so you do not need to provide them here yet.

LimeSurvey

Section D: 3. Outcomes tested for FEASIBILITY

Please rate the outcomes below on feasibility in the context of assessing the surgical learning curve of the neurosurgeon.

The outcomes below scored with consensus as sufficient for relevance in the first questionnaire round.

(23/27) CONTROL OF INSTRUMENTS

Control of instruments refers to the surgeon's ability to effectively manipulate and operate surgical tools during a procedure. This could be measured for example with machine learning-based video analysis or by supervisor assessment.

(24/27) INTRA-OPERATIVE COMPLICATIONS

Intra-operative complications are adverse events or issues that occur during a surgical procedure, which can affect the patient's safety and surgical outcomes. These complications can range from minor incidents to severe problems, such as excessive bleeding or damage to surrounding tissues resulting in neurological symptoms, and may necessitate additional interventions and should be reported in the OR report or a post-operative note.

In the first round, the most commonly cited reason for considering this outcome unfeasible was the frequent underreporting of outcomes, which could introduce bias. Additionally, it was considered necessary to create a clear list specifying which complications are included.

(25/27) EXECUTION OF THE INTENDED PLAN

Execution of the intended plan refers to the surgeon's ability to successfully carry out the preoperative surgical plan during the procedure. This outcome measure evaluates whether the surgery adhered to the intended course of action, including accuracy, technical success, efficiency and postoperative results.

(26/27) PERMANENT POST-OPERATIVE NEUROLOGICAL SYMPTOMS OR DETERIORATION

Permanent post-operative neurological symptoms or deterioration refers to the occurrence of new or worsening neurological deficits which do not resolve within 30 days following the surgical procedure and are diagnosed by the treating neurologist, oncologist, or radiation therapist.

(27/27) ADVERSE EVENTS CLAVIEN-DINDO (CD) ≥2

D1. **Do you consider it feasible to consistently measure this outcome in daily practice in the context of assessing a surgical learning curve?**

Median score (IQR) first questionnaire: 5 (4-7)

Certainly not feasible 1	2	3	4	5	6	7	8	Very feasible 9
☐	☐	☐	☐	☐	☐	☐	☐	☐

D2. **Do you consider it feasible to consistently measure this outcome in daily practice in the context of assessing a surgical learning curve?**

Median score (IQR) first questionnaire: 7 (6-9)

Certainly not feasible 1	2	3	4	5	6	7	8	Very feasible 9
☐	☐	☐	☐	☐	☐	☐	☐	☐

D3. **Do you consider it feasible to consistently measure this outcome in daily practice in the context of assessing a surgical learning curve?**

Median score (IQR) first questionnaire: 7 (5-8)

Certainly not feasible 1	2	3	4	5	6	7	8	Very feasible 9
☐	☐	☐	☐	☐	☐	☐	☐	☐

D4. **Do you consider it feasible to consistently measure this outcome in daily practice in the context of assessing a surgical learning curve?**

Median score (IQR) first questionnaire: 7 (6-8)

Certainly not feasible 1	2	3	4	5	6	7	8	Very feasible 9
☐	☐	☐	☐	☐	☐	☐	☐	☐

D5. **Do you consider it feasible to consistently measure this outcome in daily practice in the context of assessing a surgical learning curve?**

Median score (IQR) first questionnaire: 8 (6.25-8)

Certainly not feasible 1	2	3	4	5	6	7	8	Very feasible 9
☐	☐	☐	☐	☐	☐	☐	☐	☐

D6. **Comments**

Please indicate here if you have any questions or comments (for example, regarding the wording of an outcome or if you would like to see something different/more specific) about any of the above outcomes. At the end of this questionnaire, you will have the opportunity to suggest additional outcomes that should be considered in this Delphi study, so you do not need to provide them here yet.

Section E: TOP 5 AND SUGGESTION FOR OTHER OUTCOMES

E1. **TOP 5**

Choose the 5 outcomes from the list below that you consider to be the most important in the context of assessing the surgical learning curve of a neurosurgeon when operating on high-grade gliomas.

Blood loss
Procedure duration
Surgeon fatigue
Standardization of operative workflow
Length of ICU/brain care unit/recovery stay
Length of hospital stay
Initiation of adjuvant therapy <6 weeks
Transient post-operative neurological symptoms or detoration
All adverse events <72 hours
All adverse events <30 days
Adverse events CD ≥ 2 <30 days
Disease related quality of life 30 days after surgery
Overall quality of life 30 days after surgery
Post-operative infarction on DWI
Usage and control of advanced techniques
Correct positioning of the patient
Onco-functional outcome
Quality of care
Readmission rate <30 days
Reoperation rate <30 days
Mortality rate
Mortality rate <30 days
Control of instruments
Intra-operative complications
Permanent post-operative symptoms or deterioration

E2. SUGGESTIONS FOR OTHER OUTCOMES

If you have suggestions for outcomes that are not mentioned in this questionnaire but you believe are important for assessing the surgical learning curve of a neurosurgeon and should be considered in the next Delphi round, please list them below.

Section F: CLOSING QUESTIONS

F1. General Comments

Please use the space below to share any remarks about the surveyed outcomes or about this Delphi questionnaire overall.

You have reached the end of the questionnaire. Thank you for your participation in this Delphi study.
