# Supplementary material for: Core outcomes for assessing surgical learning curves in high-grade glioma surgery: a European Delphi study
Source: Brain Spine. 2026 May 16;6:106097. doi: 10.1016/j.bas.2026.106097 (PMC13397579; doi:10.1016/j.bas.2026.106097)
Supplement: Multimedia component 4 [file mmc4.pdf]

Supplementary Item 4

Core outcomes for assessing surgical learning curves in high-grade glioma surgery: a European Delphi study

Céline L.G. Neutel, MD[1], Valerie Diederen[1], Jiri Bartek, MD, PhD[2], Gerjon Hannink, PhD[3], Maroeska M. Rovers, PhD[3], Mark ter Laan, MD, PhD[1], the Expert Meeting Group[#]

[1] Department of Neurosurgery, Radboud university medical center, Nijmegen, The Netherlands.

[2] Department of Neurosurgery and Clinical Neuroscience, Karolinska University Hospital and Karolinska Institutet, Stockholm, Sweden

[3] Department of Medical Imaging, Radboud university medical center, Nijmegen, The Netherlands.

[#]

- Johnny Duerinck, MD, PhD, Department of Neurosurgery, Universitair Ziekenhuis Brussel, Vrije Universiteit Brussel, Brussels, Belgium
- Steven De Vleeschouwer, MD, PhD, Department of Neurosurgery, University Hospitals Leuven, Belgium and Department of Neurosciences, Leuven Brain Institute, KU Leuven, Belgium
- Tomas Kazda, MD, PhD, Department of Radiation oncology, Masaryk Memorial Cancer Institute, Brno, Czech Republic
- Alessia Pellerino, MD, PhD, Department of Neuroscience "Rita Levi Montalcini", University and City of Health and Science Hospital, Turin, Italy
- Michael Veldeman MD PhD, Department of Neurosurgery, RWTH Aachen University Hospital, Aachen, Germany
- Asgeir S. Jakola, MD, PhD, Institute of Neuroscience and Physiology, Department of Clinical Neuroscience, University of Gothenburg, Gothenburg, Sweden and Region Västra Götaland, Sahlgrenska University Hospital, Department of Neurosurgery, Gothenburg, Sweden
- Kostas N. Fountas, MD, PhD, Department of Neurosurgery, Faculty of Medicine, School of Health Sciences, University of Thessaly, Larisa, Greece
- Sebastian Pavel, MD, Brain Institute, Monza Hospital, Bucharest, Romania
- Dan-Andrei Mitrea, MD, Neuroaxis - Neurology Clinic, Bucharest, Romania

Supplementary Item 4

Combined results of first and second questionnaire rounds

ACCEPTED ON RELEVANCE AND FEASIBILITY		% IRRELEVANT/UNFEASIBLE	% POTENTIALLY RELEVANT/FEASIBLE	% RELEVANT/FEASIBLE
Percentage tumor resected	Relevance / feasibility	4%* / 8%*	18* / 10%*	78%* / 82%*
Residual tumor remnant	Relevance / feasibility	4%* / 6%*	20%* / 14%*	76%* / 80%*
Permanent post-operative neurological symptoms or deterioration	Relevance / feasibility	2%* / 0%	12%* / 13%	86%* / 88%
Reoperation rate <30 days after surgery	Relevance / feasibility	2% / 2%*	17% / 10%*	81% / 88%*
All adverse events <72 hours	Relevance / feasibility	6% / 2%	11% / 17%	83% / 81%
Adverse events Clavien-Dindo ≥2 <72 hours	Relevance / feasibility	2%* / 5%	18%* / 14%	80%* / 81%
Adverse events Clavien-Dindo ≥2 <30 days	Relevance / feasibility	5% / 3%	19% / 22%	77% / 75%
Usage & control of advanced techniques	Relevance / feasibility	5% / 0%	20% / 22%	75% / 78%

ACCEPTED ON RELEVANCE, NO CONSENSUS ON FEASIBILITY		% IRRELEVANT/UNFEASIBLE	% POTENTIALLY RELEVANT/FEASIBLE	% RELEVANT/FEASIBLE
Control of instruments	Relevance / feasibility	2%* / 14%	22%* / 47%	76%* / 39%
Intra-operative complications	Relevance / feasibility	0%* / 9%	16%* / 28%	84%* / 63%
Execution of the intended plan	Relevance / feasibility	4%* / 13%	18%* / 36%	78%* / 52%
Onco-functional outcome (OFO)	Relevance / feasibility	5% / 6%	11% / 30%	84% / 64%
Correct positioning of the patient	Relevance / feasibility	5% / 11%	19% / 39%	77% / 50%
Standardization of operative workflow	Relevance / feasibility	0% / 11%	25% / 50%	75% / 39%
**ACCEPTED ON FEASIBILITY, NO CONSENSUS ON RELEVANCE**		**% IRRELEVANT/UNFEASIBLE**	**% POTENTIALLY RELEVANT/FEASIBLE**	**% RELEVANT/FEASIBLE**
Readmission rate <30 days after surgery	Relevance / feasibility	5% / 2%*	25% / 8%*	70% / 90%*
Mortality rate	Relevance / feasibility	13% / 8%*	22% / 14%*	66% / 78%*
Mortality rate <30 days after surgery	Relevance / feasibility	8% / 2%*	19% / 12%*	73% / 86%*

Length of hospital stay	Relevance / feasibility	14% / 3%	47% / 16%	39% / 81%
Length of intensive care unit/brain care unit/recovery stay	Relevance / feasibility	17% / 8%	47% / 16%	36% / 77%
Initiation of adjuvant treatment <6 weeks	Relevance / feasibility	19% / 6%	42% / 16%	39% / 78%
Procedure duration	Relevance / feasibility	13% / 8%	31% / 14%	56% / 78%
Transient post-op neurological symptoms or deterioration	Relevance / feasibility	6% / 6%	25% / 17%	69% / 77%
**NO CONSENSUS ON EITHER RELEVANCE OR FEASIBILITY**		**% IRRELEVANT/UNFEASIBLE**	**% POTENTIALLY RELEVANT/FEASIBLE**	**% RELEVANT/FEASIBLE**
Blood loss	Relevance / feasibility	20% / 11%	52% / 33%	28% / 56%
Surgeon fatigue	Relevance / feasibility	17% / 38%	50% / 42%	33% / 20%
All adverse events <30 days	Relevance / feasibility	9% / 6%	39% / 31%	52% / 63%
Disease related quality of life 30 days after surgery	Relevance / feasibility	16% / 13%	30% / 47%	55% / 41%
Overall quality of life 30 days after surgery	Relevance / feasibility	16% / 11%	36% / 45%	48% / 44%

Infarction DWI post-operative	Relevance / feasibility	3% / 6%	33% / 25%	64% / 69%
Quality of care	Relevance / feasibility	6% / 11%	30% / 47%	64% / 42%

* Scores indicated with an asterisk represent outcomes that reached consensus in the first questionnaire round and were therefore directly carried over to the combined results. These outcomes were not re-evaluated in the second questionnaire round.
