# Supplementary material for: Core outcomes for assessing surgical learning curves in high-grade glioma surgery: a European Delphi study
Source: Brain Spine. 2026 May 16;6:106097. doi: 10.1016/j.bas.2026.106097 (PMC13397579; doi:10.1016/j.bas.2026.106097)
Supplement: Multimedia component 5 [file mmc5.pdf]

Supplementary Item 5

Core outcomes for assessing surgical learning curves in high-grade glioma surgery: a European Delphi study

Céline L.G. Neutel, MD[1], Valerie Diederen[1], Jiri Bartek, MD, PhD[2], Gerjon Hannink, PhD[3], Maroeska M. Rovers, PhD[3], Mark ter Laan, MD, PhD[1], the Expert Meeting Group[#]

[1] Department of Neurosurgery, Radboud university medical center, Nijmegen, The Netherlands.

[2] Department of Neurosurgery and Clinical Neuroscience, Karolinska University Hospital and Karolinska Institutet, Stockholm, Sweden

[3] Department of Medical Imaging, Radboud university medical center, Nijmegen, The Netherlands.

#

- Johnny Duerinck, MD, PhD, Department of Neurosurgery, Universitair Ziekenhuis Brussel, Vrije Universiteit Brussel, Brussels, Belgium
- Steven De Vleeschouwer, MD, PhD, Department of Neurosurgery, University Hospitals Leuven, Belgium and Department of Neurosciences, Leuven Brain Institute, KU Leuven, Belgium
- Tomas Kazda, MD, PhD, Department of Radiation oncology, Masaryk Memorial Cancer Institute, Brno, Czech Republic
- Alessia Pellerino, MD, PhD, Department of Neuroscience "Rita Levi Montalcini", University and City of Health and Science Hospital, Turin, Italy
- Michael Veldeman MD PhD, Department of Neurosurgery, RWTH Aachen University Hospital, Aachen, Germany
- Asgeir S. Jakola, MD, PhD, Institute of Neuroscience and Physiology, Department of Clinical Neuroscience, University of Gothenburg, Gothenburg, Sweden and Region Västra Götaland, Sahlgrenska University Hospital, Department of Neurosurgery, Gothenburg, Sweden
- Kostas N. Fountas, MD, PhD, Department of Neurosurgery, Faculty of Medicine, School of Health Sciences, University of Thessaly, Larisa, Greece
- Sebastian Pavel, MD, Brain Institute, Monza Hospital, Bucharest, Romania
- Dan-Andrei Mitrea, MD, Neuroaxis - Neurology Clinic, Bucharest, Romania

Supplementary Item 5

Demographic characteristics expert group

		**Expert group**
**Countries (n (%))**		
	Belgium	3 (23%)
	Czech Republic	1 (8%)
	Germany	1 (8%)
	Italy	1 (8%)
	Romania	2 (15%)
	The Netherlands	2 (15%)
	Sweden	2 (15%)
	Greece	1 (8%)
**Professions (n (%))**		
	Neurosurgeons	8 (62%)
	Neuro-oncologists	2 (15%)
	Radiation oncologists	1 (8%)
	Patients	2 (15%)
**Years of neuro-oncological experience (n (%))**		
	0 – 5 years	1 (9%)
	6 – 10 years	4 (36%)
	11 – 15 years	4 (36%)
	16 years or more	2 (18%)
**Involved in policy making (n (%))**		9 (82%)
**Academic hospitals (n (%))**		9 (82%)
**Annual # operated HGG (n (%))**		
	10 – 50	2 (18%)
	51 – 150	8 (73%)
	>150	1 (9%)
