# Supplementary material for: Core outcomes for assessing surgical learning curves in high-grade glioma surgery: a European Delphi study
Source: Brain Spine. 2026 May 16;6:106097. doi: 10.1016/j.bas.2026.106097 (PMC13397579; doi:10.1016/j.bas.2026.106097)
Supplement: Multimedia component 6 [file mmc6.pdf]

Supplementary Item 6

Core outcomes for assessing surgical learning curves in high-grade glioma surgery: a European Delphi study

Céline L.G. Neutel, MD[1], Valerie Diederen[1], Jiri Bartek, MD, PhD[2], Gerjon Hannink, PhD[3], Maroeska M. Rovers, PhD[3], Mark ter Laan, MD, PhD[1], the Expert Meeting Group[#]

[1] Department of Neurosurgery, Radboud university medical center, Nijmegen, The Netherlands.

[2] Department of Neurosurgery and Clinical Neuroscience, Karolinska University Hospital and Karolinska Institutet, Stockholm, Sweden

[3] Department of Medical Imaging, Radboud university medical center, Nijmegen, The Netherlands.

[#]

- Johnny Duerinck, MD, PhD, Department of Neurosurgery, Universitair Ziekenhuis Brussel, Vrije Universiteit Brussel, Brussels, Belgium
- Steven De Vleeschouwer, MD, PhD, Department of Neurosurgery, University Hospitals Leuven, Belgium and Department of Neurosciences, Leuven Brain Institute, KU Leuven, Belgium
- Tomas Kazda, MD, PhD, Department of Radiation oncology, Masaryk Memorial Cancer Institute, Brno, Czech Republic
- Alessia Pellerino, MD, PhD, Department of Neuroscience "Rita Levi Montalcini", University and City of Health and Science Hospital, Turin, Italy
- Michael Veldeman MD PhD, Department of Neurosurgery, RWTH Aachen University Hospital, Aachen, Germany
- Asgeir S. Jakola, MD, PhD, Institute of Neuroscience and Physiology, Department of Clinical Neuroscience, University of Gothenburg, Gothenburg, Sweden and Region Västra Götaland, Sahlgrenska University Hospital, Department of Neurosurgery, Gothenburg, Sweden
- Kostas N. Fountas, MD, PhD, Department of Neurosurgery, Faculty of Medicine, School of Health Sciences, University of Thessaly, Larisa, Greece
- Sebastian Pavel, MD, Brain Institute, Monza Hospital, Bucharest, Romania
- Dan-Andrei Mitrea, MD, Neuroaxis - Neurology Clinic, Bucharest, Romania

Supplementary Item 6

Additional details on the second expertmeeting

Following validation of the results from the two questionnaires, it was identified that one outcome, being *"Usage and control of advanced techniques"*, had not been discussed during the Expert Consensus Meeting, despite meeting the predefined criteria for discussion. To address this oversight, an additional expert meeting was organized five weeks after the initial session. This second meeting was scheduled promptly to preserve continuity and engagement among the experts.

Seven of the thirteen experts were able to attend, thereby meeting the predefined minimum required for such consensus meetings (1). Five neurosurgeons, one neuro-oncologist and one radiation oncologist from five different countries attended the additional meeting. The remaining five invitees, being three medical specialists and two patients, were unable to participate.

During this meeting, the outcome *"Usage and control of advanced techniques"* was discussed in depth. Although the experts acknowledged its relevance in the context of neurosurgical training, the outcome was excluded from the final list. Only two of the seven (29%) attending experts voted in favor of its inclusion, which did not meet the predefined threshold of 50% required for acceptance. This decision was further supported by several concerns: the wide variation in advanced techniques, which complicates consistent definition; differences in availability across countries and centers; and challenges regarding the feasibility of reliable measurement.

References

1. Kathryn Fitch SJB MDA, Bernard Burnand, Juan Ramón LaCalle, Pablo Lázaro, Mirjam van het Loo, Joseph McDonnell, John Paul Vader, James P. Kahan. The RAND/UCLA Appropriateness Method User's Manual. RAND; 2001 August 5th 2025.
