# Supplementary material for: Core outcomes for assessing surgical learning curves in high-grade glioma surgery: a European Delphi study
Source: Brain Spine. 2026 May 16;6:106097. doi: 10.1016/j.bas.2026.106097 (PMC13397579; doi:10.1016/j.bas.2026.106097)
Supplement: Multimedia component 7 [file mmc7.docx]

| **Consensus statement** | **% Out** NL+GER | **% Out** other countries | **% Maybe** NL+GER | **% Maybe** other countries | **% In**  NL+ GER | **% In**  other countries |
| --- | --- | --- | --- | --- | --- | --- |
| Procedure duration (feasibility) | 7% | 8% | 21% | 8% | 71% | 83% |
| Length of ICU/BCU/recovery stay (feasibility) | 14% | 3% | 25% | 8% | 61% | 89% |
| Length of hospital stay (feasibility) | 7% | 0% | 29% | 6% | 64% | 94% |
| Initiation of adjuvant treatment <6 weeks (feasibility) | 7% | 6% | 21% | 11% | 71% | 83% |
| Transient post-operative neurological symptoms or deterioration (feasibility) | 7% | 6% | 21% | 14% | 71% | 81% |
| All adverse events <30 days (feasibility) | 7% | 6% | 54% | 14% | 39% | 81% |
| Adverse events CD >=2 <30 days (feasibility) | 4% | 3% | 36% | 11% | 61% | 86% |
| Infarction on DWI (relevance) | 0% | 6% | 54% | 17% | 46% | 78% |
| Infarction on DWI (feasibility) | 7% | 6% | 36% | 17% | 57% | 78% |
| Usage & control advanced techniques (relevance) | 4% | 6% | 29% | 14% | 68% | 81% |
| Usage & control advanced techniques (feasibility) | 0% | 0% | 29% | 17% | 71% | 83% |
| Correct positioning of the patient (relevance) | 7% | 3% | 25% | 14% | 68% | 83% |
| Readmission rate <30 days (relevance) | 4% | 6% | 32% | 19% | 64% | 75% |
| Mortality rate <30 days (relevance) | 11% | 6% | 25% | 14% | 64% | 81% |

**Supplementary item 1:** Sensitivity analysis comparing consensus outcomes between Dutch/German respondents and all other countries in the second Delphi round.
